# Supplementary material for: Diversity-Oriented Synthesis of a Molecular Library of Immunomodulatory α-Galactosylceramides with Fluorous-Tag-Assisted Purification and Evaluation of Their Bioactivities in Regard to IL-2 Secretion
Source: Int J Mol Sci. 2022 Nov 2;23(21):13403. doi: 10.3390/ijms232113403 (PMC9659121; doi:10.3390/ijms232113403)
Supplement: Supplementary file 1 [file ijms-23-13403-s001.zip › ijms-1918519-supplementary.pdf]

Supporting Information

Diversity-Oriented Synthesis of a Molecular Library of Immunomodulatory α-Galactosylceramides with Fluorous-Tag-Assisted Purification and Evaluation of Their Bioactivities

Yeng-Nan Chen,[a,†] Jung-Tung Hung,[b,†] Fan-Dan Jan,[a] Yung-Yu Su,[a] Jih Ru Hwu,[a] Alice L. Yu,[b,c] Avijit K. Adak,[a,*] Chun-Cheng Lin [a,d,*]

[a]*Department of Chemistry, National Tsing Hua University, Hsinchu 30044, Taiwan*

[b]Institute of Stem Cell and Translational Cancer Research, Chang Gung Memorial Hospital, and Chang gung University, Linkou, Taoyuan 33302, Taiwan

[c] Department of Pediatrics, University of California, San Diego, CA 92093, USA

[d]*Department of Medicinal and Applied Chemistry, Kaohsiung Medical University, Kaohsiung 80708, Taiwan*

[*]Corresponding author: E-mail: cclin66@mx.nthu.edu.tw and akadak@mx.nthu.edu.tw
[†]Authors have equal contribution.

Table of contents

1. Materials.........S2

2. Known compounds.........S2

3. Table S1.........S3

4. Table S2.........S4

5. Synthesis of compounds.........S4

6. References.........S34

Materials. All reactions were performed in oven-dried glassware (120 °C) under a nitrogen atmosphere unless indicated otherwise. All chemicals were purchased as reagent grade and used without further purification. Dichloromethane (CH_2Cl_2) was distilled over calcium hydride. Tetrahydrofuran (THF) and ether were distilled over sodium metal/benzophenone ketyl. Anhydrous N,N-dimethylformamide (DMF) was purchased from Merck. Molecular sieves (MS) for glycosylation were MS 4Å (Aldrich) and activated by flame. ^{1}H and ^{13}C NMR spectra were recorded on either a Bruker AV-400 or AV-600 spectrometer operating at 400 or 600 MHz for ^{1}H and 100 or 150 MHz for ^{13}C, respectively. Chemical shifts (δ) are reported in ppm and referenced to the solvent used ($CDCl_3$, δ 7.24 and 77.0; CD_3OD, δ 3.31 and 49.0; D_2O, δ 4.67), with coupling constants (J) reported in Hz. Two-dimensional (COSY) experiments were used to assist in assignment of the products. High-resolution mass spectra were recorded under ESI-TOF mass spectroscopy conditions. Analytical thin-layer chromatography (TLC) was performed on pre-coated plates (silica gel 60). Silica gel 60 (E. Merck) was employed for all flash-chromatography experiments. All fluorus-assisted purification was performed using FluoroFlash® SPE cartridge (Fluorous Tech. Inc., USA). The reactions were monitored by examination under UV light (254 nm) and by staining with *p*-anisaldehyde, ninhydrin, cerium molybdate, or potassium permanganate solutions.

Known compounds. Compounds **5**,[1] **8**,[2] **17**,[2] **18**,[2] **20**,[2] **24**,[3] **27**,[4] **1a**,[5] **1b**,[6] and **1db**[7] have been previously synthesized and reported in the cited papers and their NMR spectra are in good agreement with the literature data.

Table S1. Optimization of the Witting reaction

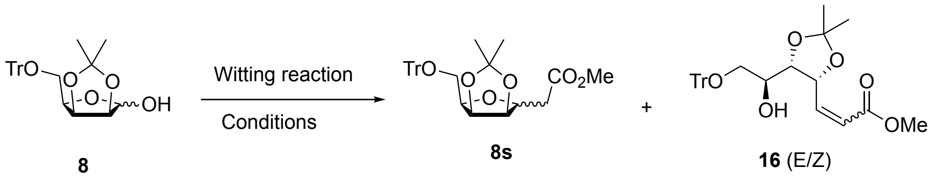

Entry	Condition	Solvent	Temperature (°C)	Product	Yield (%)
1	$(EtO)_2P(O)CH_2CO_2Et$, LiCl, DBU	CH_3CN	rt	No reaction	—
2	$(EtO)_2P(O)CH_2CO_2Et$, LiHMDS	THF	0 °C to rt	No reaction	—
3	$(EtO)_2P(O)CH_2CO_2Et$, NaH	DMSO	0 °C to rt	No reaction	—
4	$Ph_3P{=}CHCO_2Me$, Benzoic acid	Toluene	90	No reaction	—
5	$Ph_3P{=}CHCO_2Me$, Benzoic acid	CH_2Cl_2	40	No reaction	—
6	$Ph_3P{=}CHCO_2Me$, Benzoic acid	CH_3CN	40	No reaction	—
7	$Ph_3P{=}CHCO_2Me$	Toluene	90	**8s**	50
8	$Ph_3P{=}CHCO_2Me$	CH_3CN	40	**8s**	70
9	$Ph_3P{=}CHCO_2Me$	CH_3CN	80	**8s**	50
10	$Ph_3P{=}CHCO_2Me$	CH_2Cl_2	rt	*Z*-**16**	20
11	$Ph_3P{=}CHCO_2Me$	1,4-dioxane	rt	**16** (*E/Z*)	30
12	$Ph_3P{=}CHCO_2Me$	DMF	rt	**16** (*E/Z*)	30
13	$Ph_3P{=}CHCO_2Me$	DMF	80	**8s**	75
14	$Ph_3P{=}CHCO_2Me$	$CHCl_3$	rt	**16** (*E/Z*)	50
15	$Ph_3P{=}CHCO_2Me$	$CHCl_3$	50	**16** (*E/Z*)	85

Table S2. Details of the structures, yields, and the HRMS (ESI-TOF) data of **42-49**.

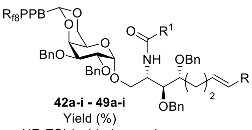 **42a-i - 49a-i** Yield (%) HR-ESI (*m/z*) observed	R = CH_2CH_3	$(CH_2)_9CH_3$	$(CH_2)_{16}CH_3$	4-ClPh	$(CH_2)_2Ph$	$(CH_2)_5Ph$	$CHCH_3(CH_2)_2CH(CH_3)_2$	$(CH_2)_24\text{-}CF_3Ph$
R¹ = C_7H_{15}	**42a** (91) (1438.5248)	—	**44a** (85) (1625.7705)	**45a** (87) (1520.4857)	**46a** (88) (1514.5560)	**47a** (85) (1556.6033)	**48a** (91) (1520.6031)	**49a** (85) (1582.5430)
$C_{25}H_{51}$	**42b** (92) (1690.8063)	**43a** (97) (1802.9323)	**44b** (93) (1091.0418)	**45b** (84) (1772.7678)	**46b** (95) (1766.8377)	**47b** (88) (1808.8849)	**48b** (90) (1565.8351)	**49b** (84) (1834.8234)
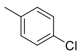	**42c** (92) (1450.4077)	—	**44c** (93) (1660.6427)	**45c** (86) (1532.3692)	**46c** (91) (1526.4390)	**47c** (89) (1568.4862)	**48c** (85) (1532.4855)	**49c** (86) (1594.4275)
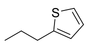	**42d** (91) (1450.4341)	—	**44d** (85) (1660.6691)	**45d** (88) (1532.3951)	**46d** (88) (1526.4652)	**47d** (92) (1568.5129)	**48d** (82) (1532.5131)	**49d** (85) (1571.4644)
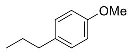	**42e** (95) (1474.4892)	—	**44e** (83) (1684.7235)	**45e** (87) (1556.4498)	**46e** (93) (1550.5197)	**47e** (91) (1592.5667)	**48e** (89) (1556.5668)	**49e** (92) (1618.5081)
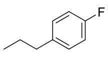	**42f** (92) (1462.4685)	—	**44f** (88) (1672.7032)	**45f** (86) (1544.4295)	**46f** (91) (1538.4998)	**47f** (85) (1580.5462)	**48f** (88) (1544.5467)	**49f** (90) (1606.4883)
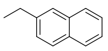	**42g** (88) (1480.4785)	—	**44g** (91) (1690.7124)	**45g** (85) (1562.4396)	**46g** (90) (1556.5097)	**47g** (83) (1598.5560)	**48g** (93) (1562.5563)	**49g** (90) (1624.4973)
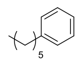	**42h** (92) (1482.5250)	—	**44h** (92) (1696.7590)	**45h** (92) (1568.4857)	**46h** (93) (1562.5559)	**47h** (87) (1604.6030)	**48h** (90) (1568.6029)	**49h** (93) (1630.5427)
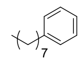	**42i** (90) (1514.5554)	**43b** (88) (1626.6811)	**44i** (93) (1724.7911)	**45i** (92) (1596.5177)	**46i** (92) (1590.5875)	**47i** (89) (1632.6340)	**48i** (89) (1996.6345)	**49i** (88) (1658.5746)

Synthesis of compounds

General procedure for the final deprotection of compounds 42-49. The fully unprotected glycolipid library was constructed by two deprotection steps: the hydrolysis of ^{F}benzylidene acetal was performed using CSA following a similar method as described in the synthesis of compound **30**, and subjected to reductive hydrogenolysis to remove benzyl ethers and reduce unsaturation in the phytosphingosine chain. In a typical experiment, CSA (1.0 eq.) was added to a solution of corresponding 4,6-*O*-^{F}benzylidene derivative (1 eq.) in MeOH/$CHCl_3$ (1/1, 2 mL) at rt with vigorous stirring. After being stirred for 8 h, the reaction mixture was quenched with Et_3N, concentrated under reduced pressure, and used without further purification. To a solution of above crude product in MeOH/CH_2Cl_2 (1/1, 2 mL), 10% $Pd(OH)_2$ (10 w/w %) was added. The resultant mixture was degassed and saturated with balloon filled with H_2 gas and left stirring at rt for 8 h under a positive pressure of H_2. The catalyst was removed by filtration through celite, rinsed with MeOH. The combined filtrates were evaporated *in vacuo*, and

subjected to flash column chromatography on silica to afford pure α-GalCer glycolipids.

Compound 1bb. R_f 0.4 (DCM/MeOH = 6/1); $[\alpha]_D^{28}$ = +24.77 (c = 2.82, MeOH/DCM = 1/1); ^{1}H NMR (600 MHz, MeOD): δ 0.90 (t, *J* = 7.0 Hz, 3H), 0.91 (t, *J* = 6.8 Hz, 3H), 1.22-1.66 (m, 56H), 2.22 (t, *J* = 7.5 Hz, 2H, H-1”), 3.55 (m, 1H), 3.60 (t, *J* = 6.2 Hz, 1H). 3.67-3.71 (m, 3H), 3.74 (dd, *J* = 3.2, 10.1 Hz, 1H), 3.78 (dd, *J* = 3.7, 10.1 Hz, 1H), 3.83 (t, *J* = 6.0 Hz, 1H), 3.87-3.90 (m, 2H), 4.21 (dd, *J* = 4.6, 10.4 Hz, 1H), 4.87 (1H merged with water); ^{13}C NMR (150 MHz, MeOD): δ 14.55, 23.85, 23.87, 27.12, 27.22, 30.56, 30.57, 30.67, 30.80 (2C), 30.85 (18C), 33.18, 33.24, 37.42, 52.03, 62.93, 68.49, 70.42, 71.23, 71.71, 72.77, 73.15, 75.74, 101.35, 176.07; HRMS (ESI-TOF) *m/z* calcd for $C_{42}H_{83}NO_9Na$ $[M+Na]^+$ 768.5960, found 768.5952.

Compound 1bc. R_f 0.4 (DCM/MeOH = 6/1); $[\alpha]_D^{28}$ = +38.62 (c = 2.28, MeOH/DCM = 1/1); 1H NMR (600 MHz, MeOD): δ 0.87 (t, *J* = 6.6 Hz, 3H), 1.25-1.44 (m, 10H), 3.62 (ddd, *J* = 2.5, 6.7, 9.2 Hz, 1H), 3.67 (dd, *J* = 5.1, 11.4, 1H), 3.70 (dd, *J* = 5.4, 11.4 Hz, 1H), 3.73-3.85 (m, 5H), 3.87 (d, *J* = 2.9 Hz, 1H), 4.03 (dd, *J* = 4.6, 10.1 Hz, 1H), 4.47 (td, *J* = 5.1, 5.4 Hz, 1H), 4.93 (d, *J* = 3.8 Hz, 1H), 7.45-7.85 (m, 4H); ^{13}C NMR (150 MHz, MeOD): δ 14.52, 23.82, 26.97, 30.54, 33.14, 33.49, 52.87, 62.97, 68.29, 70.39, 71.27, 71.72, 72.78, 73.19, 75.82, 101.37, 128.57 (2C), 129.69 (2C), 132.83, 135.99, 170.12; HRMS (ESI-TOF) *m/z* calcd for $C_{23}H_{36}ClNO_9Na$ $[M+Na]^+$ 528.1971, found 528.1964.

Compound 1bd. R_f 0.4 (DCM/MeOH = 6/1); $[\alpha]_D^{30}$ = +33.16 (c = 5.03, MeOH/DCM = 1/1); ^{1}H NMR (400 MHz, MeOD): δ 0.88 (t, *J* = 6.7 Hz, 3H), 1.26-1.67 (m, 10H), 2.57, (t, *J* = 7.1 Hz, 2H), 3.13, (t, *J* = 7.1 Hz, 2H), 3.46-3.79 (m, 8H), 3.85 (dd, *J* = 4.6, 10.8 Hz, 1H), 3.89 (d, *J* = 2.7 Hz, 1H), 4.21 (td, *J* = 4.7, 9.7 Hz, 1H), 4.86 (d, *J* = 3.7 Hz, 1H), 6.82-7.14 (m, 3H); ^{13}C NMR (100 MHz, MeOD): δ 14.51, 23.52, 26.07, 26.64, 30.32, 32.83, 33.31, 38.88, 51.56, 62.63, 67.99, 69.90, 70.79, 71.21, 72.04, 72.74, 75.53, 100.81, 124.23, 125.52, 127.63, 144.21, 173.90; HRMS (ESI-TOF) *m/z* calcd for $C_{23}H_{36}ClNO_9Na$ $[M+Na]^+$ 528..2238, found 528.2240.

Compound 1be. R_f 0.4 (DCM/MeOH = 6/1); $[\alpha]_D^{30}$ = +33.16 (c = 5.03, MeOH/DCM = 1/1); ^{1}H NMR (400 MHz, MeOD): δ 0.88 (t, *J* = 6.7 Hz, 3H), 1.26-1.67 (m, 10H), 2.57, (t, *J* = 7.1 Hz, 2H), 3.13, (t, *J* = 7.1 Hz, 2H), 3.46-3.79 (m, 8H), 3.85 (dd, *J* = 4.6, 10.8 Hz, 1H), 3.89 (d, *J* = 2.7 Hz, 1H), 4.21 (td, *J* = 4.7, 9.7 Hz, 1H), 4.86 (d, *J* = 3.7 Hz, 1H), 6.82-7.14 (m, 3H); ^{13}C NMR (100 MHz, MeOD): δ 14.51, 23.52, 26.07, 26.64, 30.32, 32.83, 33.31, 38.88, 51.56, 62.63, 67.99, 69.90, 70.79, 71.21, 72.04, 72.74, 75.53, 100.81, 124.23, 125.52, 127.63, 144.21, 173.90; HRMS (ESI-TOF) *m/z* calcd for $C_{23}H_{36}ClNO_9Na$ $[M+Na]^+$ 528..2238, found 528.2240.

Compound 1bf . R_f 0.4 (DCM/MeOH = 6/1); $[\alpha]_D^{28}$ = +39.21 (c = 2.21, MeOH/DCM = 1/1); ^{1}H NMR (400 MHz, MeOD): δ 0.88 (t, *J* = 6.8 Hz, 3H), 1.26-1.64 (m, 10H), 2.49 (t, *J* = 7.7 Hz, 2H, H-1”), 2.88 (t, *J* = 7.7 Hz, 2H, H-2”), 3.46 (m, 1H), 3.50 (t, *J* = 6.0 Hz, 1H), 3.61 (dd, *J* = 4.8, 10.8 Hz, 1H), 3.66-3.79 (m, 5H), 3.81 (dd, *J* = 4,6, 10.8 Hz, 1H), 3.85 (d, *J* = 2.5 Hz, 1H), 4.18 (td, *J* = 4.8, 10.1 Hz, 1H), 4.82 (d, *J* = 3.7 Hz, 1H), 6.95-6.99 (m, 2H), 7.19-7.22 (m, 2H); ^{13}C NMR (100 MHz, MeOD): δ 14.59, 23.86, 26.99, 30.66, 32.10, 33.19, 33.41, 39.04, 51.98, 62.91, 68.26, 70.33, 71.18, 71.64, 72.74, 73.02, 75.82, 101.25, 116.07, 131.16, 131.24, 138.28, 161.80, 164.21, 174.84; HRMS (ESI-TOF) *m/z* calcd for $C_{25}H_{40}FNO_9Na$ $[M+Na]^+$ 540.2579, found 540.2573.

Compound 1bh. R_f 0.4 (DCM/MeOH = 6/1); $[\alpha]_D^{27}$ = +38.48 (c = 2.19, MeOH/DCM = 1/1); ^{1}H NMR (600 MHz, MeOD): δ 0.90 (t, *J* = 5.9 Hz, 3H), 1.18-1.73 (m, 16H), 2.22 (t, *J* = 7.4 Hz, 2H, H-1”), 2.61 (t, *J* = 7.7 Hz, 2H, H-5”), 3.53-3.74 (m, 6H), 3.79 (dd, *J* = 3.5, 10.1, 1H), 3.83 (t, *J* = 6.0 Hz, 1H), 3.88 (d, *J* = 3.6 Hz, 1H), 3.89 (dd, *J* = 4.1, 10.5 Hz, 1H), 4.22 (td, *J* = 4,9, 10.1 Hz, 1H), 4.87 (d, *J* = 3.5 Hz, 1H), 7.12-7.25 (m, 5H); ^{13}C NMR (150 MHz, MeOD): δ 14.57, 23.86, 27.03, 27.09, 30.09, 30.66, 32.57, 33.22, 33.40, 36.86, 37.33, 52.02, 62.93, 68.45, 70.40, 71.23, 71.71, 72.76, 73.15, 75.87, 101.33, 126.81, 129.42 (2C), 129.54 (2C), 143.94, 175.96; HRMS (ESI-TOF) *m/z* calcd for $C_{28}H_{47}NO_9Na$ $[M+Na]^+$ 564.3143, found 564.3138.

Compound 1bi. R*f* 0.4 (DCM/MeOH = 6/1); $[\alpha]_D^{27}$ = +33.84 (c = 2.21, MeOH/DCM = 1/1); ^{1}H NMR (600 MHz, MeOD): δ 0.90 (t, *J* = 6.8 Hz, 3H), 1.16-1.72 (m, 20H), 2.22 (t, *J* = 7.5 Hz, 2H, H-1"), 2.60 (t, *J* = 7.7 Hz, 2H, H-7"), 3.53-3.75 (m, 6H), 3.79 (dd, *J* = 3.5, 10.0 Hz, 1H), 3.83 (t, *J* = 5.9 Hz, 1H), 3.89 (m, 2H, H), 4.21 (td, *J* = 4,7, 10.7 Hz, 1H), 4.87 (d, *J* = 3.5 Hz, 1H), 7.11-7.25 (m, 5H); ^{13}C NMR (150 MHz, MeOD): δ 14.58, 23.86, 27.10, 27.68, 30.47, 30.66, 31.10 32.85, 33.22, 34.77, 37.04, 37.38, 38.84, 39.15, 52.02, 62.93, 68.47, 70.40, 71.23, 71.71, 72.75, 73.14, 75.82, 101.32, 126.76, 129.39 (2C), 129.52 (2C), 144.10, 176.03; HRMS (ESI-TOF) *m/z* calcd for $C_{30}H_{51}NO_9Na$ $[M+Na]^+$ 592.3456, found 592.3453.

Compound 1ca. R*f* 0.4 (DCM/MeOH = 6/1); $[\alpha]_D^{29}$ = +16.31 (c = 2.23, MeOH/DCM = 1/1); ^{1}H NMR (800 MHz, MeOD/DCM = 1/1): δ 0.85 (t, *J* = 6.4 Hz, 3H), 0.86 (t, *J* = 6.2 Hz, 3H), 1.24-1.64 (m, 50H), 2.19 (t, *J* = 7.6 Hz, 2H, H-1"), 3.52-3.74 (m, 6H, H-3, H-6a, H-1', H-3', H-4',), 3.77 (dd, *J* = 3.8, 10.1 Hz, 1H), 3.79 (d, *J* = 6.0 Hz, 1H), 3.86 (dd, *J* = 4.6, 10.8 Hz, 1H), 3.90 (d, *J* = 2.6 Hz, 1H), 4.18 (td, *J* = 4.7, 9.8 Hz, 1H), 4.87 (d, *J* = 3.8 Hz, 1H), 7.11-7.24 (m, 10H); ^{13}C NMR (200 MHz, MeOD / DCM = 1 / 1): δ 14.43, 14.46, 23.31, 23.35, 26.58, 26.63, 29.78, 29.99, 30.05, 30.34, 30.37 (7C), 30.40 (4C), 30.42, 30.46, 30.49, 32.44, 32.63, 33.06, 37.09, 51.25, 62.47, 67.95, 69.1, 70.54, 71.03, 71.73, 72.64, 75.29, 100.55, 175.38; HRMS (ESI-TOF) *m/z* calcd for $C_{39}H_{77}NO_9Na$ $[M+Na]^+$ 703.5598, found 703.5592.

Compound 1cb. R*f* 0.5 (DCM/MeOH = 6/1); $[\alpha]_D^{29}$ = +7.53 (c = 1.91, MeOH/DCM = 1/1); ^{1}H NMR (400 MHz, MeOD/DCM = 1/1): δ 0.85 (t, *J* = 6.8 Hz, 6H), 1.16-1.66 (m, 82H), 2.18 (t, *J* = 7.3 Hz, 2H, H-1"), 3.51-3.74 (m, 6H, H-3, H-6a, H-1', H-3', H-4',), 3.77 (dd, *J* = 3.7, 9.9 Hz, 1H), 3.78 (t, *J* = 5.7 Hz, 1H), 3.85 (dd, *J* = 4.5, 10.7 Hz, 1H), 3.90 (d, *J* = 3.0 Hz, 1H), 4.16 (td, *J* = 4.6, 9.5 Hz, 1H), 4.87 (d, *J* = 3.7 Hz, 1H), 7.11-7.24 (m, 10H); ^{13}C NMR (200 MHz, MeOD/DCM = 1/1): δ 13.31, 13.34, 22.19, 22.23, 25.46, 25.51, 28.66, 28.88, 28.93, 29.21, 29.25 (18C), 29.30 (12C), 29.34, 29.37, 31.32, 31.51, 31.94, 35.97, 50.13, 61.35, 66.83, 68.59, 69.42, 69.91, 70.61, 71.52, 74.17, 99.43, 174.26; HRMS (ESI-TOF) *m/z* calcd for

$C_{57}H_{113}NO_9Na$ [M+Na]$^+$ 955.8415, found 955.8421.

Compound 1cc. R_f 0.4 (DCM/MeOH = 6/1); $[\alpha]_D^{29}$ = +19.69 (c = 1.89, MeOH/DCM = 1/1); ^{1}H NMR (400 MHz, MeOD): δ 0.90 (t, *J* = 6.8 Hz, 3H), 1.25-1.69 (m, 40H), 3.60 (m, 1H), 3.64-3.83 (m, 7H), 3.86 (d, *J* = 3.4 Hz, 1H), 4.0 (dd, *J* = 4.4, 10.9 Hz, 1H), 4.44 (dd, *J* = 4.9, 10.8 Hz, 1H), 4.89 (1H merge with water), 7.46-7.86 (m, 4H); ^{13}C NMR (100 MHz, MeOD): δ 14.46, 23.32, 26.39, 26.45, 30.02, 30.35 (14C), 32.60, 33.22, 51.93, 62.47, 67.74, 69.65, 70.53, 71.0, 71.67, 72.65, 75.28, 100.52, 127.89 (2C), 129.19 (2C), 132.41, 134.95, 169.31; HRMS (ESI-TOF) *m/z* calcd for $C_{38}H_{66}ClNO_9Na$ [M+Na]$^+$ 738.4318, found 738.4312.

Compound 1cd. R_f 0.4 (DCM/MeOH = 6/1); $[\alpha]_D^{29}$ = +22.31 (c = 1.76, MeOH/DCM = 1/1); ^{1}H NMR (400 MHz, MeOD): δ 0.90 (d, *J* = 6.8 Hz, 3H), 1.29-1.67 (m, 40H), 2.37 (dt, *J* = 2.4, 7.4 Hz, 2H, H-1”), 3.14 (t, *J* = 7.4 Hz, 2H, H-2”), 3.50 (m, 1H), 3.56 (t, *J* = 6.1 Hz, 1H), 3.64-3.82 (m, 6H), 3.86 (dd, *J* = 4.2, 10.5 Hz, 1H), 3.87 (d, *J* = 4.1 Hz, 1H), 4.20 (dd, *J* = 4.7, 10.4 Hz, 1H), 4.85 (d, *J* = 3.7 Hz, 1H), 6.84-7.18 (m, 3H); ^{13}C NMR (200 MHz, MeOD): δ 14.60, 23.89, 26.41, 26.93, 27.07, 30.63, 30.92 (8C), 30.98 (6C), 33.23, 33.30, 39.17, 52.04, 62.92, 68.29, 70.35, 71.20, 71.63, 72.74, 73.00, 75.74, 101.30, 124.54, 125.86, 127.95, 144.73, 175.26; HRMS (ESI-TOF) *m/z* calcd for $C_{38}H_{69}NO_9SNa$ [M+Na]$^+$ 738.4585, found 738.4577.

Compound 1ce. R_f 0.4 (DCM/MeOH = 6/1); $[\alpha]_D^{29}$ = +25.38 (c = 2.15, MeOH/DCM = 1/1); ^{1}H NMR (400 MHz, MeOD): δ 0.84 (d, *J* = 6.1 Hz, 3H), 1.23-1.61 (m, 40H), 2.49 (t, *J* = 7.7 Hz, 2H, H-1”), 2.86 (t, *J* = 7.7 Hz, 2H, H-2”), 3.47-3.79 (m, 11H, H-2, H-3, H-5, H-6a, H-1’, H-3’, H-4’, OMe), 3.83 (dd, *J* = 4.6, 10.8 Hz, 1H), 3.87 (d, *J* = 2.6 Hz, 1H), 4.20 (td, *J* = 4.7, 10.1 Hz, 1H), 4.85 (1H merge with water), 6.82-7.14 (m, 4H); ^{13}C NMR (100 MHz, MeOD): δ 14.52, 23.50, 26.76, 29.60, 29.86, 30.21, 30.53 (12C), 32.47, 32.79, 33.06, 37.18, 41.28, 51.47, 55.94, 62.57, 68.05, 69.87, 70.70, 71.19, 72.02, 72.75, 75.35, 100.75, 114.71, 129.35 (2C), 130.09 (2C), 158.50, 175.58; HRMS (ESI-TOF) *m/z* calcd for $C_{41}H_{73}NO_{10}Na$ [M+Na]$^+$ 762.5127, found 762.5123.

Compound 1cf. R_f 0.4 (DCM/MeOH = 6/1); $[\alpha]_D^{29}$ = +24.44 (c = 2.01, MeOH/DCM = 1/1); ^{1}H NMR (400 MHz, MeOD): δ 0.87 (d, *J* = 6.7 Hz, 3H), 1.25-1.63 (m, 40H), 2.49 (t, *J* = 7.7 Hz, 2H, H-1”), 2.89 (t, *J* = 7.7 Hz, 2H, H-2”), 3.47-3.79 (m, 8H), 3.81 (dd, *J* = 4.6, 10.8 Hz, 1H), 3.88 (d, *J* = 3.0 Hz, 1H), 4.18 (td, *J* = 5.0, 9.6 Hz, 1H), 4.85 (d, *J* = 3.6 Hz, 1H), 6.93-7.20 (m, 4H); ^{13}C NMR (200 MHz, MeOD): δ 14.48, 23.41, 26.57, 30.12, 30.40, 30.44 (9C), 30.48 (4C), 30.57, 31.67, 32.70, 33.22, 38.72, 51.38, 62.53 67.91, 69.76, 70.60, 71.09, 71.88, 72.62, 75.40, 100.64, 115.78, 115.88, 130.57, 130.61, 137.39, 61.77, 162.98, 174.11; HRMS (ESI-TOF) *m/z* calcd for $C_{40}H_{70}FNO_9Na$ [M+Na]$^+$ 750.4927, found 750.4924.

Compound 1cg. R_f 0.4 (DCM/MeOH = 6/1); $[\alpha]_D^{29}$ = +26.93 (c = 1.85, MeOH/DCM = 1/1); ^{1}H NMR (400 MHz, MeOD): δ 0.90 (d, *J* = 6.8 Hz, 3H), 1.16-1.57 (m, 40H), 3.49 (m, 1H)3.55-3.72 (m, 10H), 3.72 (s, 2H, H-1”), 3.75 (dd, *J* = 3.9, 10.1Hz, 1H), 3.87 (dd, *J* = 4.2, 10.6 Hz, 1H), 4.19 (dd, *J* = 4.4, 10.6 Hz, 1H), 4.84 (d, *J* = 3.9 Hz, 1H), 7.43-7.84 (m, 7H); ^{13}C NMR (100 MHz, MeOD): δ 14.22, 22.88, 26.03, 29.56, 29.86 (2C), 29.91 (7C), 29.94 (6C), 32.14, 32.96, 43.26, 50.68, 62.22, 69.14, 70.04, 70.47, 70.81, 70.93, 72.41, 74.93, 99.84, 125.94, 126.35, 129.75 (2C), 129.94, 130.01, 130.10, 131.99, 136.64, 137.86, 172.64; HRMS (ESI-TOF) *m/z* calcd for $C_{43}H_{71}NO_9Na$ [M+Na]$^+$ 768.5021, found 768.5017.

Compound 1ch. R_f 0.4 (DCM/MeOH = 6/1); $[\alpha]_D^{29}$ = +23.51 (c = 2.12, MeOH/DCM = 1/1); ^{1}H NMR (400 MHz, MeOD/$CDCl_3$ = 1/1): δ 0.86 (d, *J* = 6.7 Hz, 3H), 1.16-1.69 (m, 46H), 2.20 (t, *J* = 7.5 Hz, 2H, H-1”), 2.59 (t, *J* = 7.6 Hz, 2H, H-5”), 3.47-3.82 (m, 8H), 3.86 (dd, *J* = 4.3, 10.6 Hz, 1H), 3.90 (d, *J* = 3.3 Hz, 1H), 4.17 (dd, *J* = 5.2, 9.6 Hz, 1H), 4.87 (d, *J* = 3.4 Hz, 1H), 6.95-7.24 (m, 5H); ^{13}C NMR (100 MHz, MeOD/$CDCl_3$ = 1/1): δ 14.39, 23.16, 26.38, 26.92, 29.85 (2C), 30.20 (14C), 32.43, 32.97, 33.06, 33.94, 37.90, 38.23, 51.01, 62.34, 67.86, 69.47, 70.33, 70.80, 71.38, 72.53, 75.15, 100.27, 126.16, 128.75 (2C), 128.84 (2C), 143.02,

Compound 1ci. R_f 0.4 (DCM/MeOH = 6/1); $[\alpha]_D^{29}$ = +18.37 (c = 1.71, MeOH/DCM = 1/1);

^{1}H NMR (400 MHz, MeOD/$CDCl_3$ = 1/1): δ 0.82 (d, *J* = 6.8 Hz, 3H), 1.25-1.69 (m, 50H), 2.19 (t, *J* = 7.5 Hz, 2H, H-1"), 2.49 (t, *J* = 7.6 Hz, 2H, H-7"), 3.48-3.82 (m, 8H), 3.86 (dd, *J* = 4.9, 10.8 Hz, 1H, H,H-6b), 3.90 (d, *J* = 3.3 Hz, 1H), 4.17 (td, *J* = 5.2, 9.6 Hz, 1H), 4.87 (d, *J* = 3.4 Hz, 1H), 6.92-7.24 (m, 5H); ^{13}C NMR (100 MHz, MeOD/$CDCl_3$ = 1/1): δ 14.55, 23.61, 26.76, 26.88, 27.40, 27.70, 30.28, 30.33, 30.64 (14C), 32.30, 32.59, 32.92, 34.46, 38.44, 38.81, 51.63, 62.67, 69.15, 70.04, 70.86, 71.34, 72.24, 72.84, 73.56, 75.30, 100.91, 127.16, 129.14 (2C), 129.17 (2C), 143.80, 175.71; HRMS (ESI-TOF) *m/z* calcd for $C_{45}H_{81}NO_9Na$ $[M+Na]^+$ 802.5804, found 802.5800.

Compound 1da. R_f 0.4 (DCM/MeOH = 6/1); $[\alpha]_D^{29}$ = +46.69 (c = 2.09, MeOH/DCM = 1/1); ^{1}H NMR (600 MHz, MeOD): δ 0.90 (t, *J* = 7.0 Hz, 3H), 1.19-1.74 (m, 16H), 2.21 (t, *J* = 7.7 Hz, 2H, H-1"), 2.62 (t, *J* = 7.5 Hz, 2H), 3.53-3.75 (m, 7H), 3.78 (dd, *J* = 3.8, 10.0 Hz, 1H). 3.82 (t, *J* = 5.9 Hz, 1H), 3.87 (d, *J* = 4.4 Hz, 1H), 3.88 (dd, *J* = 4.4, 10.6 Hz, 1H), 4.21 (td, *J* = 4.8, 10.7 Hz, 1H), 4.87 (d, *J* = 3.8 Hz, 1H), 7.11-7.24 (m, 5H); ^{13}C NMR (150 MHz, MeOD): δ 14.54, 23.83, 26.76, 27.20, 27.69, 30.30, 30.51, 32.96, 33.05, 37.11, 37.41, 52.02, 62.94, 68.49, 70.41, 71.24, 71.71, 72.78, 73.01, 75.81, 101.35, 126.75, 129.39 (2C), 129.55 (2C), 144.13, 176.04; HRMS (ESI-TOF) *m/z* calcd for $C_{28}H_{47}NO_9Na$ $[M+Na]^+$ 564.3143, found 564.3135.

Compound 1db. R_f 0.4 (DCM/MeOH = 6/1); $[\alpha]_D^{29}$ = +29.95 (c = 2.35, MeOH/DCM = 1/1); ^{1}H NMR (600 MHz, MeOD): δ 0.90 (t, *J* = 6.6 Hz, 3H), 1.25-1.77 (m, 52H), 2.20 (t, *J* = 7.6 Hz, 2H, H-1"), 2.59 (t, *J* = 7.4 Hz, 2H), 3.61-3.85 (m, 8H), 3.87 (d, *J* = 2.6 Hz, 1H), 4.02 (dd, *J* = 4.5, 10.9 Hz, 1H), 4.46 (td, *J* = 4.9, 10.8 Hz, 1H), 4.93 (d, *J* = 3.8 Hz, 1H), 7.10-7.24 (m, 5H); ^{13}C NMR (150 MHz, MeOD): δ 14.55, 23.85, 26.48 26.62, 27.68, 30.29, 30.58 (2C), 30.86 (17C), 32.84, 33.16, 33.19, 37.01, 52.89, 62.98, 68.35, 70.40, 71.26, 71.72, 72.81, 73.06, 75.78, 101.40, 126.70, 129.36 (2C), 129.54 (2C), 144.11, 170.15; HRMS (ESI-TOF) *m/z* calcd for $C_{46}H_{83}NO_9Na$ $[M+Na]^+$ 816.5960, found 816.5954.

Compound 1dc. R_f 0.4 (DCM/MeOH = 6/1); $[\alpha]_D^{29}$ = +52.20 (c = 2.0, MeOH/DCM = 1/1); ^1H NMR (600 MHz, MeOD): δ 1.43-1.78 (m, 6H), 2.59 (t, *J* = 7.4 Hz, 2H), 3.61-3.85 (m, 8H), 3.86 (d, *J* = 2.9 Hz, 1H). 4.02 (dd, *J* = 4.5, 10.8 Hz, 1H), 4.46 (td, *J* = 4.9, 10.6 Hz, 1H), 4.93 (d, *J* = 3.7 Hz, 1H), 7.11-7.85 (m, 9H); ^{13}C NMR (150 MHz, MeOD): δ 26.63, 30.87, 32.85, 37.02, 52.89, 62.98, 68.34, 70.41, 71.26, 71.73, 72.82, 73.06, 75.78, 101.40 126.71, 128.58 (2C), 129.36 (2C), 129.54 (2C), 129.71 (2C), 132.83, 136.00, 144.11, 170.15; HRMS (ESI-TOF) *m/z* calcd for $C_{27}H_{36}ClNO_9Na$ $[M+Na]^+$ 576.1971, found 576.1970.

Compound 1dd. R_f 0.4 (DCM/MeOH = 6/1); $[\alpha]_D^{29}$ = +48.79 (c = 2.28, MeOH/DCM = 1/1); ^1H NMR (600 MHz, MeOD): δ 1.55-1.71 (m, 6H), 2.50 (dt, *J* = 3.4, 7.7 Hz, 2H, 1"), 2.61 (t, *J* = 7.9 Hz, 2H), 2.88 (t, *J* = 7.7 Hz, 2H, H-2"), 3.48-3.80 (m, 8H), 3.85 (dd, *J* = 4.5, 10.7 Hz, 1H), 3.88 (d, *J* = 5.0 Hz, 1H), 4.20 (td, *J* = 4.8, 10.3 Hz, 1H), 4.84 (d, *J* = 3.8 Hz, 1H), 6.96-7.24 (m, 8H); ^{13}C NMR (150 MHz, MeOD): δ 26.66, 32.10, 32.97, 33.20, 37.09, 39.05, 52.04, 62.96, 68.39, 70.38, 71.23, 71.68, 72.77, 72.93, 75.86, 101.32, 126.75, 129.39 (2C), 129.55 (2C), 131.15, 131.21, 134.14, 138.30, 144.14, 174.76; HRMS (ESI-TOF) *m/z* calcd for $C_{27}H_{39}NO_9SNa$ $[M+Na]^+$ 576.2238, found 576.2229.

Compound 1de. R_f 0.4 (DCM/MeOH = 6/1); $[\alpha]_D^{29}$ = +48.38 (c = 2.0, MeOH/DCM = 1/1); ^1H NMR (600 MHz, MeOD): δ 1.55-1.71 (m, 6H), 2.48 (t, *J* = 7.7 Hz, 2H, H-1"), 2.61 (t, *J* = 7.7 Hz, 2H), 2.84 (t, *J* = 7.7 Hz, 2H, H-2"), 3.48-3.80 (m, 11H, , H-2, H-3, H-5, H-6a, H-1', H-3', H-4', OMe), 3.83 (dd, *J* = 4.8, 10.7 Hz, 1H), 3.86 (d, *J* = 3.2 Hz, 1H), 4.19 (td, *J* = 5.0, 10.1 Hz, 1H), 4.87 (d, *J* = 3.7 Hz, 1H), 6.81-7.24 (m, 9H); ^{13}C NMR (150 MHz, MeOD): δ 26.68, 32.14, 32.98, 33.20, 37.09, 39.33, 52.03, 55.85, 62.98, 68.39, 70.40, 71.24, 71.68, 72.77, 72.94, 75.88, 101.34, 115.08 (2C), 126.75, 129.40 (2C), 129.56 (2C), 130.47 (2C), 134.33, 144.16, 159.75, 175.09; HRMS (ESI-TOF) *m/z* calcd for $C_{30}H_{43}NO_{10}Na$ $[M+Na]^+$ 600.2779, found 600.2777.

Compound 1df. R_f 0.4 (DCM/MeOH = 6/1); $[\alpha]_D^{29}$ = +46.90 (c = 2.15, MeOH/DCM = 1/1);

^{1}H NMR (600 MHz, MeOD): δ 1.56-1.73 (m, 6H), 2.21 (t, *J* = 7.6 Hz, 2H, H-1”), 2.61 (t, *J* = 7.5 Hz, 2H), 2.84 (t, *J* = 7.6 Hz, 2H, H-2”), 3.51-3.88 (m, 8H), 3.88 (d, *J* = 3.9 Hz, 1H), 3.89 (dd, *J* = 4.2, 10.7 Hz, 1H), 4.21 (td, *J* = 4.9, 10.6 Hz, 1H), 4.87 (d, *J* = 3.7 Hz, 1H), 6.81-7.24 (m, 9H); ^{13}C NMR (150 MHz, MeOD): δ 26.75, 30.21, 32.84, 32.96, 37.11, 39.32, 52.01, 62.94, 68.48, 70.40, 71.2, 71.70, 72.75, 72.99, 75.81, 101.33, 115.08 (2C), 126.74, 129.38 (2C), 129.55 (2C), 130.46 (2C), 134.32, 144.13, 159.74, 176.04; HRMS (ESI-TOF) *m/z* calcd for $C_{29}H_{40}FNO_9Na$ $[M+Na]^+$ 588.2579, found 588.2584.

Compound 1dh. R_f 0.4 (DCM/MeOH = 6/1); $[\alpha]_D^{28}$ = +44.79 (c = 2.10, MeOH/DCM = 1/1); ^{1}H NMR (400 MHz, MeOD): δ 1.15-1.70 (m, 12H), 2.21 (t, *J* = 7.3 Hz, 2H, H-1”), 2.59 (t, *J* = 7.56 Hz, 2H), 2.61 (t, *J* = 7.3 Hz, 2H, H-2”), 3.53-3.84 (m, 8H), 3.87 (d, *J* = 3.7 Hz, 1H), 3.88 (dd, *J* = 4.2, 9.9 Hz, 1H), 4.21 (dd, *J* = 5.0, 9.6 Hz, 1H), 4.85 (1H merged with water), 7.11-7.26 (m, 10H); ^{13}C NMR (100 MHz, MeOD): δ 27.68, 30.09, 30.88, 32.98, 34.74 36.87, 37.12, 37.30, 39.14, 51.97, 62.91, 68.41, 70.37, 71.20, 71.66, 72.76, 72.93, 75.69, 101.30, 126.75, 126.81, 129.39 (2C), 129.42 (2C), 129.55 (4C), 143.93, 144.10, 175.95; ; HRMS (ESI-TOF) *m/z* calcd for $C_{29}H_{40}FNO_9Na$ $[M+Na]^+$ 588.2579, found 588.2584.

Compound 1ea. R_f 0.4 (DCM/MeOH = 6/1); $[\alpha]_D^{30}$ = +46.70 (c = 1.94, MeOH/DCM = 1/1); ^{1}H NMR (400 MHz, MeOD/$CDCl_3$ = 1/1): δ 0.85 (t, *J* = 6.6 Hz, 3H), 1.13-1.67 (m, 20H), 2.18 (t, *J* = 7.6 Hz, 2H, H-1”), 2.57 (t, *J* = 7.7 Hz, 2H, H-10’), 3.52-3.80 (m, 8H), 3.86 (dd, *J* = 4.6, 10.7 Hz, 1H). 3.90 (d, *J* = 2.0 Hz, 1H), 4.19 (td, *J* = 3.9, 9.2 Hz, 1H), 4.87 (d, *J* = 3.6 Hz, 1H), 7.12-7.23 (m, 5H); ^{13}C NMR (100 MHz, MeOD/$CDCl_3$ = 1/1): δ 14.41, 23.28, 26.60, 27.12, 29.74, 29.97, 30.29, 32.26, 32.41, 33.08, 34.17, 36.61, 37.08, 51.22, 62.46 67.92, 69.68, 70.52, 71.02, 71.70, 72.59, 75.33, 100.53, 126.20, 128.84 (2C), 129.01 (2C), 143.52, 175.36; HRMS (ESI-TOF) *m/z* calcd for $C_{30}H_{51}NO_9Na$ $[M+Na]^+$ 592.3456, found 592.3450.

Compound 1eb. R_f 0.4 (DCM/MeOH = 6/1); $[\alpha]_D^{30}$ = +24.48 (c = 2.85, MeOH/DCM = 1/1); ^{1}H NMR (400 MHz, MeOD/$CDCl_3$ = 1/1): δ 0.87 (t, *J* = 6.8 Hz, 3H), 1.25-1.69 (m, 56H),

2.20 (t, J = 7.6 Hz, 2H, H-1”), 2.58 (t, J = 7.7 Hz, 2H, H-10’), 3.53-3.82 (m, 8H), 3.86 (dd, J = 4.3, 10.6 Hz, 1H). 3.89 (d, J = 3.16 Hz, 1H), 4.18 (td, J = 3.8, 10.0 Hz, 1H), 4.87 (d, J = 3.6 Hz, 1H), 7.11-7.24 (m, 5H); ^{13}C NMR (100 MHz, MeOD/$CDCl_3$ = 1/1): δ 14.53, 23.53, 26.70, 26.86, 30.24 (2C), 30.33, 30.53 (18C), 32.51, 32.83, 33.02, 34.40, 36.81, 37.22, 51.51, 62.60, 68.08, 69.94, 70.74, 71.24, 72.09, 72.74, 75.38, 100.79, 126.39, 129.03 (2C), 129.19 (2C), 143.71, 175.62; HRMS (ESI-TOF) *m/z* calcd for $C_{48}H_{87}NO_9Na$ $[M+Na]^+$ 844.6273, found 844.6277.

Compound 1ec. R_f 0.4 (DCM/MeOH = 6/1); $[\alpha]_D^{30}$ = +43.87 (c = 1.89, MeOH/DCM = 1/1); ^{1}H NMR (400 MHz, MeOD/$CDCl_3$ = 1/1): δ 1.11-1.71 (m, 10H), 2.54 (t, J = 7.7 Hz, 2H, H-10’), 3.58-3.82 (m, 8H), 3.88 (d, J = 3.0 Hz, 1H), 3.99 (dd, J = 4.7, 10.8 Hz, 1H), 4.43 (td, J = 4.8, 9.7 Hz, 1H), 4.93 (d, J = 3.7 Hz, 1H), 7.11-7.24 (m, 5H); ^{13}C NMR (100 MHz, MeOD/$CDCl_3$ = 1/1): δ 26.41, 29.99, 30.24, 32.28, 34.20, 36.60, 52.03, 62.53, 67.78, 69.74, 70.59, 71.09, 71.83, 72.64, 75.35, 100.63 126.22, 127.95 (2C), 128.87 (2C), 129.05 (2C), 129.25 (2C), 132.45, 135.06, 143.58, 169.38; HRMS (ESI-TOF) *m/z* calcd for $C_{29}H_{40}ClNO_9Na$ $[M+Na]^+$ 604.2284, found 604.2277.

Compound 1ee. R_f 0.4 (DCM/MeOH = 6/1); $[\alpha]_D^{30}$ = +39.34 (c = 2.01, MeOH/DCM = 1/1); ^{1}H NMR (400 MHz, MeOD/$CDCl_3$ = 1/1): δ 1.17-1.63 (m, 10H), 2.47 (t, J = 7.8 Hz, 2H, H-1”), 2.57 (t, J = 7.7 Hz, 2H, H-10’), 2.84 (t, J = 7.7 Hz, 2H, H-2”), 3.47-3.76 (m, 11H, H-2, H-3, H-5, H-6a, H-1’, H-3’, H-4’, OMe), 3.80 (dd, J = 4.8, 10.9 Hz, 1H), 3.88 (d, J = 2.8 Hz, 1H), 4.16 (m, 1H), 4.84 (d, J = 3.6 Hz, 1H), 6.78-7.23 (m, 9H); ^{13}C NMR (200 MHz, MeOD/$CDCl_3$ = 1/1): δ 26.39, 29.98, 30.29, 31.60, 32.29, 33.07, 36.59, 38.87, 51.30, 55.59, 62.43, 67.76, 69.69, 70.52, 71.00, 71.83, 72.46, 75.31, 100.56, 114.54 (2C), 126.18, 128.83 (2C), 128.99 (2C), 129.92 (2C), 133.60, 143.52, 158.88, 174.38; HRMS (ESI-TOF) *m/z* calcd for $C_{32}H_{47}NO_{10}Na$ $[M+Na]^+$ 628.3092, found 628.3088.

Compound 1ef. R_f 0.4 (DCM/MeOH = 6/1); $[\alpha]_D^{30}$ = +44.59 (c = 2.06, MeOH/DCM = 1/1);

^{1}H NMR (400 MHz, MeOD/CDCl$_3$ = 1/1): δ 1.25-1.59 (m, 10H), 2.20 (t, *J* = 7.6 Hz, 2H, H-1"), 2.57 (t, *J* = 7.7 Hz, 2H, H-10'), 2.79 (t, *J* = 7.6 Hz, 2H, H-2"), 3.52-3.82 (m, 8H), 3.87 (dd, *J* = 4.7, 10.9 Hz, 1H), 3.89 (d, *J* = 3.0 Hz, 1H), 4.19 (td, *J* = 4.7, 9.7 Hz, 1H), 4.87 (d, *J* = 3.6 Hz, 1H), 6.92-7.42 (m, 9H); ^{13}C NMR (100 MHz, MeOD/CDCl$_3$ = 1/1): δ 26.74, 30.29, 30.61, 31.92, 32.66, 33.27, 36.90, 38.87, 51.68, 62.73, 68.07, 70.06, 70.90, 71.37, 72.35, 72.77, 75.58, 100.96, 115.90, 116.11, 126.51, 129.15 (2C), 129.31 (2C), 130.86, 130.93, 137.84, 143.86, 161.49, 163.91, 174.45; HRMS (ESI-TOF) *m/z* calcd for $C_{31}H_{44}FNO_9$ Na [M+Na]$^+$ 616.2892, found 616.2883.

Compound 1eg. R$_f$ 0.4 (DCM/MeOH = 6/1); $[\alpha]_D^{29}$ = +26.91 (c = 2.15, MeOH/DCM = 1/1); ^{1}H NMR (400 MHz, MeOD/CDCl$_3$ = 1/1): δ 1.20-1.63 (m, 10H), 2.55 (t, *J* = 7.7 Hz, 2H), 3.43 (s, 2H), 3.44-3.89 (m, 8H, H-2, H-3, H-5, H-6a, H-1', H-3', H-4', H-1"), 3.82 (d, *J* = 2.4 Hz, 1H), 3.88 (m, 1H), 4.14 (td, *J* = 4.4, 9.0 Hz, 1H), 4.83 (d, *J* = 3.8 Hz, 1H), 6.93-7.23 (m, 12H); ^{13}C NMR (100 MHz, MeOD/CDCl$_3$ = 1/1): δ 26.24, 29.52, 29.84, 30.10, 32.06, 36.46, 43.47, 51.05, 62.37, 67.73, 69.44, 70.33, 70.77, 71.28, 72.53, 75.06, 100.23, 126.04, 126.11, 126.63, 128.70 (2C), 128.86 (2C), 129.33, 129.55, 130.01, 130.35, 132.43, 136.25, 136.55, 136.71, 143.37, 173.07; HRMS (ESI-TOF) *m/z* calcd for $C_{34}H_{45}NO_9$ Na [M+Na]$^+$ 634.2987, found 634.2984.

Compound 1eh. R$_f$ 0.4 (DCM/MeOH = 6/1); $[\alpha]_D^{29}$ = +34.93 (c = 1.73, MeOH/DCM = 1/1); ^{1}H NMR (400 MHz, MeOD/CDCl$_3$ = 1/1): δ 1.25-1.67 (m, 16H), 2.19 (t, *J* = 7.5 Hz, 2H, H-1"), 2.56 (t, *J* = 7.5 Hz, 2H, H-10'), 2.58 (t, *J* = 7.5 Hz, 2H, H-5"), 3.50-3.80 (m, 8H), 3.86 (dd, *J* = 4.8, 10.9 Hz, 1H), 3.88 (d, *J* = 3.0 Hz, 1H), 4.19 (td, *J* = 4.7, 9.7 Hz, 1H), 4.87 (d, *J* = 3.7 Hz, 1H), 7.11-7.24 (m, 10H); ^{13}C NMR (100 MHz, MeOD/CDCl$_3$ = 1/1): δ 26.59, 26.63, 29.71, 30.15, 30.43, 32.10, 32.44, 33.14, 36.51, 36.74, 37.07, 51.43, 62.56, 68.02, 69.86, 70.67, 71.19, 72.00, 72.70, 75.43, 100.74, 126.33, 126.44, 128.98 (2C), 129.03 (2C), 129.14 (4C), 143.37, 143.66, 175.42; HRMS (ESI-TOF) *m/z* calcd for $C_{34}H_{51}NO_9$ Na [M+Na]$^+$ 640.3456,

found 640.3452.

Compound 1ei. R_f 0.4 (DCM/MeOH = 6/1); $[\alpha]_D^{29}$ = +31.69 (c = 1.94, MeOH/DCM = 1/1); ^{1}H NMR (400 MHz, MeOD/$CDCl_3$ = 1/1): δ 1.25-1.60 (m, 20H), 2.19 (t, *J* = 7.5 Hz, 2H, H-1”), 2.57 (t, *J* = 7.5 Hz, 4H, H-10’, H-7”), 3.52-3.81 (m, 8H), 3.86 (dd, *J* = 4.6,10.7 Hz, 1H), 3.89 (d, *J* = 2.2 Hz, 1H), 4.18 (td, *J* = 4.4, 9.2 Hz, 1H), 4.87 (d, *J* = 3.6 Hz, 1H), 7.09-7.24 (m, 10H); ^{13}C NMR (100 MHz, MeOD/$CDCl_3$ = 1/1): δ 26.61, 26.72, 29.93, 30.07, 30.13, 30.41, 32.36, 32.41, 33.07, 34.30, 36.69, 36.72, 37.13, 51.39, 62.55, 68.01, 69.84, 70.65, 71.17, 71.95, 72.68, 75.39, 100.70, 126.32, 126.35, 128.96 (2C), 128.98 (2C), 129.12 (4C), 143.57, 143.64, 175.47; HRMS (ESI-TOF) *m/z* calcd for $C_{36}H_{55}NO_9$ Na $[M+Na]^+$ 668.3769, found 668.3766.

Compound 1fa. R_f 0.4 (DCM/MeOH = 6/1); $[\alpha]_D^{29}$ = +33.70 (c = 2.04, MeOH/DCM = 1/1); ^{1}H NMR (400 MHz, MeOD): δ 0.87 (t, *J* = 6.7 Hz, 3H), 1.11-1.69 (m, 26H), 2.19 (t, *J* = 7.6 Hz, 2H, H-1”), 2.57 (t, *J* = 7.7 Hz, 2H, H-13’), 3.52-3.82 (m, 8H), 3.86 (dd, *J* = 4.7, 10.9 Hz, 1H), 3.89 (d, *J* = 2.6 Hz, 1H), 4.18 (td, *J* = 4.6, 9.6 Hz, 1H), 4.87 (d, *J* = 3.6 Hz, 1H), 7.11-7.24 (m, 5H); ^{13}C NMR (100 MHz, MeOD): δ 14.42, 23.30, 26.56, 26.61, 29.76, 29.98, 30.22, 30.31, 30.40, 30.45, 32.29, 32.42, 33.06, 34.18, 36.63, 37.08, 51.23, 62.46, 67.93, 69.69, 70.52, 71.02, 71.71, 72.62, 75.29 100.53, 126.19, 128.85 (2C), 129.02 (2C), 143.58, 175.37; HRMS (ESI-TOF) *m/z* calcd for $C_{33}H_{57}NO_9Na$ $[M+Na]^+$ 634.3926, found 634.3925.

Compound 1fb. R_f 0.4 (DCM/MeOH = 6/1); $[\alpha]_D^{29}$ = +22.53 (c = 1.88, MeOH/DCM = 1/1); ^{1}H NMR (400 MHz, MeOD): δ 0.86 (t, *J* = 6.8 Hz, 3H), 1.35-1.69 (m, 62H), 2.19 (t, *J* = 7.5 Hz, 2H, H-1”), 2.56 (t, *J* = 7.8 Hz, 2H, H-10’), 3.53-3.79 (m, 8H), 3.86 (dd, *J* = 4.4, 10.5 Hz, 1H), 3.89 (m, 1H), 4.17 (m, 1H), 4.87 (d, *J* = 3.3 Hz, 1H), 7.12-7.24 (m, 5H); ^{13}C NMR (200 MHz, MeOD): δ 14.40, 23.20, 26.41, 26.46, 29.89 (2C), 29.99, 30.11, 30.18 (2C), 30.21 (12C), 30.24 (4C), 30.30, 30.34, 32.06, 32.47, 32.97, 34.03, 36.47, 37.00, 51.06, 62.37, 67.90, 69.53, 70.36, 70.86, 71.44, 72.54, 75.28, 100.33, 126.07, 128.72 (2C), 128.89 (2C), 143.39, 175.22; HRMS (ESI-TOF) *m/z* calcd for $C_{51}H_{93}NO_9Na$ $[M+Na]^+$ 886.6743, found 886.6741.

Compound 1fc. R*f* 0.4 (DCM/MeOH = 6/1); $[\alpha]_D^{29}$ = +29.24 (c = 2.05, MeOH/DCM = 1/1); ^{1}H NMR (400 MHz, MeOD): δ 1.26-1.72 (m, 16H), 2.57 (t, *J* = 7.7 Hz, 2H, H-10'), 3.60 (m, 1H), 3.65-3.84 (m, 7H), 3.89 (d, *J* = 2.8 Hz, 1H), 4.0 (dd, *J* = 4.5, 10.9 Hz, 1H), 4.43 (dd, *J* = 4.9, 10.5 Hz, 1H), 4.92 (d, *J* = 3.8 Hz, 1H), 7.10-7.24 (m, 5H), 7.44 (d, *J* = 8.9 Hz, 2H), 7.81 (d, *J* = 8.6 Hz, 2H); ^{13}C NMR (100 MHz, MeOD/$CDCl_3$ = 1/1): δ 16.96, 17.66, 26.77, 30.34, 30.55, 30.68, 32.74, 33.17, 36.96, 52.72, 62.80, 68.04, 70.15, 71.00, 71.45, 72.53, 72.88, 75.41, 101.13, 126.57, 129.21, 129.36 (2C), 129.46 (2C), 129.69 (2C), 130.11 (2C), 132.20, 143.94, 168.66; HRMS (FAB) *m/z* calcd for $C_{32}H_{46}ClNO_9Na$ $[M+Na]^+$ 646.2753, found 646.2755.

Compound 1fd. R*f* 0.4 (DCM/MeOH = 6/1); $[\alpha]_D^{29}$ = +27.88 (c = 1.50, MeOH/DCM = 1/1); ^{1}H NMR (400 MHz, MeOD): δ 1.26-1.63 (m, 16H), 2.57 (t, *J* = 7.5 Hz, 4H, H-1", H-13'), 3.13 (t, *J* = 7.5 Hz, 2H, H-2"), 3.46-3.80 (m, 8H), 3.84 (dd, *J* = 4.5,10.8 Hz, 1H), 3.88 (d, *J* = 2.8 Hz, 1H), 4.17 (m, 1H), 4.85 (d, *J* = 3.7 Hz, 1H), 6.82-7.24 (m, 8H); ^{13}C NMR (100 MHz, MeOD): δ 16.97, 17.66, 26.34, 26.83, 26.92, 30.39, 30.65, 30.76, 32.79, 36.99, 39.08, 51.87, 62.82, 68.20, 70.20, 71.03, 71.48, 72.48, 72.90, 75.67, 101.13, 124.43, 125.74, 126.62, 127.84, 129.26 (2C), 129.42 (2C), 143.99, 144.53, 174.15; HRMS (ESI-TOF) *m/z* calcd for $C_{32}H_{49}NO_9SNa$ $[M+Na]^+$ 646.3020, found 646.3023.

Compound 1fe. R*f* 0.4 (DCM/MeOH = 6/1); $[\alpha]_D^{29}$ = +35.87 (c = 1.67, MeOH/DCM = 1/1); ^{1}H NMR (400 MHz, MeOD): δ 1.11-1.66 (m, 16H), 2.45 (t, *J* = 7.7 Hz, 2H), 2.54 (t, *J* = 7.7 Hz, 2H), 2.82 (t, *J* = 7.7 Hz, 2H), 3.46-3.84 (m, 12H, H-2, H-3, H-5, H-6, H-1', H-3', H-4', OMe), 3.87 (d, *J* = 2.2 Hz, 1H), 4.15 (m, 1H), 4.82 (d, *J* = 3.7 Hz, 1H), 6.77-7.21 (m, 9H); ^{13}C NMR (100 MHz, MeOD/$CDCl_3$ = 1/1): δ 27.24, 27.55, 30.10, 30.46, 30.55, 31.76, 32.42, 34.30, 36.73, 38.59, 39.01, 51.42, 55.73, 62.58, 67.92, 69.81, 70.66, 71.13, 71.92, 72.64, 75.43, 100.68, 114.67 (2C), 126.30, 128.95 (2C), 129.12 (2C), 130.04 (2C), 133.74, 143.68, 159.00, 174.50; HRMS (ESI-TOF) *m/z* calcd for $C_{35}H_{53}NO_{10}Na$ $[M+Na]^+$ 670.3562, found 670.3557.

Compound 1ff. R*f* 0.4 (DCM/MeOH = 6/1); $[\alpha]_D^{29}$ = +34.23 (c = 2.11, MeOH/DCM = 1/1);

^{1}H NMR (400 MHz, MeOD): δ 1.29-1.66 (m, 16H), 2.51 (t, *J* = 7.5 Hz, 2H, H-13'), 2.58 (t, *J* = 7.7 Hz, 2H, H-1"), 2.90 (t, *J* = 7.7 Hz, 2H, H-2"), 3.47-3.80 (m, 8H), 3.84 (dd, *J* = 4.6, 11.0 Hz, 1H), 3.87 (d, *J* = 2.4 Hz, 1H),4.20 (td, *J* = 4.7, 9.7 Hz, 1H), 4.85 (d, *J* = 3.7 Hz, 1H), 6.96-7.25 (m, 9H); ^{13}C NMR (100 MHz, MeOD): δ 27.03, 30.49, 30.75, 30.87, 30.90, 30.95, 32.13, 32.93, 33.32, 37.07, 39.05, 51.96, 62.91, 68.24, 70.32, 71.19, 71.62, 72.76, 72.96, 75.75, 101.26, 116.07, 116.29, 126.73, 129.37 (2C), 129.51 (2C), 131.144, 131.22, 138.29, 144.13, 161.80, 164.21, 174.74; HRMS (ESI-TOF) *m/z* calcd for $C_{34}H_{50}FNO_9Na$ $[M+Na]^+$ 658.3362, found 658.3357.

Compound 1fg. R*f* 0.4 (DCM/MeOH = 6/1); $[\alpha]_D^{28}$ = +23.21 (c = 2.13, MeOH/DCM = 1/1); ^{1}H NMR (400 MHz, MeOD): δ 1.19-1.63 (m, 16H), 2.54 (t, *J* = 7.7 Hz, 2H, H-13'), 3.42-3.89 (m, 10H, H-2, H-3, H-5, H-6a, H-1', H-3', H-4', H-1"), 3.82 (d, *J* = 2.4 Hz, 1H), 3.86 (m, 1H), 4.14 (td, *J* = 4.4, 9.0 Hz, 1H), 4.83 (d, *J* = 3.8 Hz, 1H), 6.91-7.24 (m, 12H); ^{13}C NMR (100 MHz, MeOD/$CDCl_3$ = 1/1): δ 26.22, 27.80, 29.52, 29.80, 29.84, 30.10, 30.18, 32.06, 36.46, 43.47, 51.05, 62.37, 67.73, 69.44, 70.33, 70.77, 71.28, 72.53, 75.06, 99.73, 125.74, 126.01, 126.53, 128.64 (2C), 128.78 (2C), 129.33, 129.55, 130.01, 130.35, 132.43, 136.25, 136.55, 136.71, 143.37, 174.17; HRMS (ESI-TOF) *m/z* calcd for $C_{37}H_{51}NO_9$ Na $[M+Na]^+$ 676.3456, found 676.3455.

Compound 1fh. R*f* 0.4 (DCM/MeOH = 6/1); $[\alpha]_D^{28}$ = +27.92 (c = 2.03, MeOH/DCM = 1/1); ^{1}H NMR (400 MHz, MeOD/$CDCl_3$ = 1/1): δ 1.14-1.69 (m, 22H), 2.20 (t, *J* = 7.5 Hz, 2H, H-13'), 2.56 (t, *J* = 7.7 Hz, 2H, H-1"), 2.59 (t, *J* = 7.7 Hz, 2H, H-2"), 3.52-3.82 (m, 8H), 3.86 (dd, *J* = 4.9, 11.1 Hz, 1H), 3.89 (d, *J* = 2.3 Hz, 1H), 4.19 (m, 1H), 4.87 (d, *J* = 3.6 Hz, 1H), 7.11-7.24 (m, 10H); ^{13}C NMR (200 MHz, MeOD/$CDCl_3$ = 1/1): δ 26.47, 27.16, 29.62, 30.01, 30.34, 30.43, 30.49, 31.99, 32.32, 33.11, 34.21, 36.42, 36.65, 36.99 51.28, 62.48, 67.96, 69.72, 70.56, 71.05, 71.77, 72.65, 75.33, 100.57, 126.22, 126.35, 128.88 (2C), 128.94 (2C), 129.04 (2C), 129.05 (2C), 143.24, 143.61, 175.28; HRMS (ESI-TOF) *m/z* calcd for $C_{37}H_{57}NO_9Na$

$[M+Na]^+$ 682.3926, found 682.3923.

Compound 1fi. R_f 0.4 (DCM/MeOH = 6/1); $[\alpha]_D^{29}$ = +25.77 (c = 2.0, MeOH/DCM = 1/1); ^1H NMR (400 MHz, MeOD/$CDCl_3$ = 1/1): δ 1.14-1.69 (m, 26H), 2.20 (t, *J* = 7.4 Hz, 2H, H-1”), 2.56 (t, *J* = 7.1 Hz, 2H, H-13’), 2.59 (t, *J* = 7.4 Hz, 2H, H-7”), 3.53-3.82 (m, 8H), 3.86 (dd, *J* = 4.6, 10.9 Hz, 1H), 3.89 (m, 1H), 4.19 (m, 1H), 4.87 (d, *J* = 3.2 Hz, 1H), 7.11-7.24 (m, 10H); ^{13}C NMR (100 MHz, MeOD/$CDCl_3$ = 1/1): δ 26.69, 26.81, 29.81, 30.05 30.20, 30.46, 30.55, 30.66, 30.80, 32.22, 32.56, 34.42, 36.60, 36.83, 37.23, 51.55, 62.63 68.14, 69.98, 70.79, 71.29, 72.15, 72.80, 75.37, 100.84, 126.41, 126.53, 129.06, 129.11, 129.22, 143.47, 143.77, 175.64; HRMS (ESI-TOF) *m/z* calcd for $C_{39}H_{61}NO_9Na$ $[M+Na]^+$ 710.4239, found 710.4231.

Compound 1ga. R_f 0.4 (DCM/MeOH = 6/1); $[\alpha]_D^{29}$ = +17.52 (c = 1.64, MeOH/DCM = 1/1); ^1H NMR (400 MHz, MeOD/$CDCl_3$ = 1/1): δ 0.87 (t, *J* = 6.8 Hz, 3H), 1.26-1.35 (m, 14H), 1.56-1.67 (m, 6H), 2.20 (t, *J* = 7.6 Hz, 2H, H-1”), 2.67 (t, *J* = 7.8 Hz, 2H, H-10’), 3.52-3.83 (m, 8H), 3.87 (dd, *J* = 4.0, 10.3 Hz, 1H), 3.89 (d, *J* =2.7 Hz, 1H), 4.19 (td, *J* =4.7, 10.0 Hz, 1H), 4.88 (d, *J* = 3.7 Hz, 1H), 7.31 (d, *J* = 7.6 Hz, 2H), 7.50 (d, *J* = 8.0 Hz, 2H); ^{13}C NMR (100 MHz, MeOD/$CDCl_3$ = 1/1) δ 14.66, 23.71, 26.87, 27.10, 30.20, 30.32, 30.40, 30.61, 32.36, 32.87, 33.15, 36.77, 37.39, 51.72, 62.81, 68.25, 70.17, 70.79, 71.49, 72.41, 72.89, 75.60, 101.06, 126.14, 129.93 (4C), 130.94, 148.47, 175.81; HRMS (FAB) *m/z* calcd for $C_{31}H_{50}NO_9F_3Na$ $[M + Na]^+$ 660.3335, found 660.3339.

Compound 1gb. R_f 0.5 (DCM/MeOH = 6/1); $[\alpha]_D^{29}$ = +8.33 (c = 0.88, MeOH/DCM = 1/1); ^1H NMR (400 MHz, MeOD/$CDCl_3$ = 1/1): δ 0.84 (t, *J* = 6.7, 3H), 1.18-1.31 (m, 50H), 1.53-1.62 (m, 6H), 2.17 (t, *J* = 7.6 Hz, 2H, H-1”), 2.63 (t, *J* = 7.7 Hz, 2H, H-10’), 3.52 (m, 2H), 3.64 (dd, *J* = 2.8, 11.1 Hz, 1H), 3.67-3.77 (m, 5H), 3.84 (dd, *J* = 4.5, 10.7 Hz, 1H), 3.89 (d, *J* = 2.8 Hz, 1H), 4.16 (td, *J* = 4.4, 8.8 Hz, 1H), 4.87 (d, *J* = 3.6 Hz, 1H), 7.26 (d, *J* = 7.9 Hz, 2H), 7.46 (d, *J* = 8.0 Hz, 2H, merge with $CDCl_3$); ^{13}C NMR (100 MHz, MeOD/$CDCl_3$ = 1/1): δ 14.28, 23.06, 26.14, 26.32, 29.64, 29.76, 29.84, 29.97, 30.08 (18C), 31.61, 32.33, 32.77, 36.14, 36.85,

50.90, 62.18, 67.66, 69.37, 70.16, 70.70 71.33, 72.29, 75.05, 100.19, 125.48, 129.14 (4C), 132.62, 147.52, 175.04; HRMS (FAB) *m/z* calcd for $C_{48}H_{86}NO_9F_3Na$ $[M + Na]^+$ 912.6147, found 912.6149.

Compound 1gc. R_f 0.4 (DCM/MeOH = 6/1); $[\alpha]_D^{29}$ = +23.36 (c = 2.05, MeOH/DCM = 1/1); ^1H NMR (400 MHz, MeOD): δ 1.29-1.47 (m, 4H), 1.56-1.62 (m, 3H), 1.68-1.70 (m, 1H), 2.64 (d, *J* = 7.8 Hz, 2H, H-10'), 3.59-3.63 (m, 1H), 3.66-3.84 (m, 7H), 3.86 (d, *J* = 3.3 Hz, 1H), 4.01 (dd, *J* = 4.4, 10.8 Hz, 1H), 4.44 (dd, *J* = 5.0, 10.7 Hz, 1H), 4.92 (d, *J* = 3.8 Hz, 1H, H-1, merge with water), 7.32 (d, *J* = 8.0 Hz, 2H), 7.45-7.48 (m, 2H), 7.53 (d, *J* = 8.0 Hz, 2H), 7.82-7.85 (m, 2H); ^{13}C NMR (100 MHz, MeOD); δ 26.67, 30.19, 30.37, 32.33, 32.96, 36.60, 52.84, 62.79, 68.04, 70.21, 71.06, 71.50, 72.70, 72.81, 75.39, 101.23, 126.11, 129.71 (2C), 130.00 (2C), 130.21 (4C), 134.46, 138.76, 148.74, 168.73. HRMS (FAB) *m/z* calcd for $C_{30}H_{39}NO_9F_3ClNa$ $[M + Na]^+$ 672.2163, found 672.2166.

Compound 1gd. R_f 0.4 (DCM/MeOH = 6/1); $[\alpha]_D^{29}$ = +25.11 (c = 2.32, MeOH/DCM = 1/1); ^1H NMR (400 MHz, MeOD/$CDCl_3$ = 1/1): δ 1.35-1.67 (m, 10H), 2.59 (dt, *J* = 2.7, 7.3 Hz, 2H, H-1"), 2.70 (t, *J* = 7.7 Hz, 2H, H-10'), 3.14 (d, *J* = 7.3 Hz, 2H, H-2"), 3.50 (m, 1H), 3.54-3.73 (m, 5H), 3.78 (d, *J* = 3.6, 10.1 Hz, 1H). 3.80 (m, 1H), 3.86 (dd, *J* = 4.6, 11.0 Hz, 1H), 3.87 (d, *J* = 4.0 Hz, 1H), 4.21 (td, *J* = 4.7, 10.3 Hz, 1H), 4.85 (d, *J* = 3.6 Hz, 1H), 6.83-6.84 (m, 1H), 6.88-6.90 (m, 1H), 7.16 (d, *J* = 5.7 Hz, 1H), 7.36 (d, *J* = 8.0 Hz, 2H), 7.54 (d, *J* = 8.0 Hz, 2H). ^{13}C NMR (100 MHz, MeOD) δ 26.81 (2C), 30.30, 30.60, 32.41, 33.13, 36.69, 39.03, 51.90, 62.79, 68.18, 70.23, 71.08, 71.50, 72.61, 72.84, 75.63, 101.18, 124.41, 125.73, 126.13, 127.82, 130.06 (4C), 133.08, 144.59, 148.77, 174.19. HRMS (FAB) *m/z* calcd for $C_{30}H_{42}NO_9F_4SNa$ $[M + Na]^+$ 672.2430, found 672.2435.

Compound 1ge. R_f 0.4 (DCM/MeOH = 6/1); $[\alpha]_D^{29}$ = +22.71 (c = 1.54, MeOH/DCM = 1/1); ^1H NMR (400 MHz, MeOD/$CDCl_3$ = 1/1) δ 1.27-1.35 (m, 6H), 1.52-1.53 (m, 1H), 1.64-1.66 (m, 3H), 2.48 (t, *J* = 7.7 Hz, 2H, H-1"), 2.68 (t, *J* = 7.7 Hz, 2H, H-2"), 2.85 (t, *J* = 7.7 Hz, 2H,

H-10’), 3.47-3.53 (m, 2H), 3.63 (dd, *J* = 4.7, 10.7 Hz, 1H), 3.68-3.79 (m, 5H), 3.76 (s, 3H, OMe), 3.82 (dd, *J* = 4.7, 10.1 Hz, 1H), 3.87 (d, *J* = 2.9 Hz, 1H), 4.19 (dd, *J* = 4.8, 9.9 Hz, 1H), 4.83 (1H merged with water peak), 6.80 (d, *J* = 8.53 Hz, 2H), 7.11 (d, *J* = 8.5 Hz, 2H), 7.32 (d, *J* = 8.2 Hz, 2H), 7.51 (d, *J* = 8.2 Hz, 2H). ^{13}C NMR (100 MHz, MeOD/$CDCl_3$ = 1/1) δ 26.59, 30.11, 30.41, 31.86, 32.18, 33.01, 36.54, 39.06, 51.59, 55.61, 62.63, 67.96, 69.98, 70.82 71.27, 72.28, 72.60, 75.44, 100.89, 114.74 (2C), 125.95, 129.78 (2C), 130.14 (4C), 133.88, 148.39, 159.28, 174.69; HRMS (FAB) *m/z* calcd for $C_{79}H_{77}NO_{11}F_{20}Na$ $[M+Na]^+$ 1618.5075, found 1618.5081.

Compound 1gf. R$_f$ 0.4 (DCM/MeOH = 6/1); $[\alpha]_D^{29}$ = +23.62 (c = 1.05, MeOH/DCM = 1/1); ^{1}H NMR (400 MHz, MeOD/$CDCl_3$ = 1/1): δ 1.25-1.65 (m, 10H), 2.49 (t, *J* = 7.7, 2H, H-1”), 2.66 (t, *J* = 7.7 Hz, 2H, H-2”), 2.88 (t, *J* = 7.7 Hz, 2H, H-10’), 3.47-3.52 (m, 2H), 3.63 (dd, *J* = 4.5, 10.9 Hz, 1H), 3.67-3.83 (m. 6H, H-2, H-3, H5), 3.88 (d, *J* = 2.7 Hz, 1H), 4.18 (m, 1H), 4.84 (d, *J* = 3.7 Hz, 1H), 6.93 (d, *J* = 8.8 Hz, 1H), 6.96 (d, *J* = 8.7 Hz, 1H), 7.16 (d, *J* = 5.4 Hz, 1H), 7.18 (d, *J* = 5.5 Hz, 1H), 7.30 (d, *J* = 8. Hz, 2H), 7.49 (d, *J* = 8.1 Hz, 2H). ^{13}C NMR (100 MHz, MeOD/$CDCl_3$ = 1/1) δ 26.78, 30.29, 30.62, 32.01, 32.45, 33.30, 36.75, 38.99, 51.75, 62.82, 68.15, 70.13, 70.98, 71.45, 72.40, 72.83, 75.66, 101.03, 116.00, 116.22, 124.44, 126.30, 128.86, 129.18, 129.94, 130.93, 131.01, 137.85, 137.88, 148.48, 161.55, 163.97, 174.53. HRMS (FAB) calcd for $C_{32}H_{43}NO_9F_4Na$ $[M + Na]^+$ 684.2772, found 684.2770.

Compound 1gg. R$_f$ 0.4 (DCM/MeOH = 6/1); $[\alpha]_D^{29}$ = +20.52 (c = 3.61, MeOH/DCM = 1/1); ^{1}H NMR (400 MHz, MeOD/$CDCl_3$ = 1/1) δ 1.16-1.57 (m, 10H), 2.61 (t, *J* = 7.6 Hz, 2H, H-10’), 3.49 (ddd, *J* = 2.3, 5.9, 9.5 Hz, 1H), 3.56-3.70 (m, 7H), 3.71 (brs, 2H, H-1”), 3.73 (d, *J* = 4.8 Hz, 1H), 3.76 (dd, *J* = 3.8, 10.0 Hz, 1H), 3.87 (dd, *J* = 4.1, 10.6 Hz, 1H), 4.19 (td, *J* = 4.3, 6.5 Hz, 1H), 4.85 (d, *J* = 3.8 Hz, 1H), 7.32 (d, *J* = 7.8 Hz, 2H), 7.43-7.48 (m, 3H), 7.54 (d, *J* = 7.8 Hz, 2H), 7.79-7.82 (m, 4H). ^{13}C NMR (100 MHz, MeOD/$CDCl_3$ = 1/1) δ 26.76, 30.20, 30.45, 32.26, 32.74, 36.63, 44.23, 52.65, 62.83, 68.31, 70.18, 71.01, 71.44, 72.55, 72.89, 75.28,

101.12, 126.07, 126.81, 127.24, 128.31, 128.68, 128.71, 128.85, 129.31, 130.02 (4C), 133.90, 134.44, 135.01, 148.80, 173.44. HRMS (FAB) calcd for $C_{35}H_{44}NO_9F_3Na$ $[M+Na]^+$ 702.2866, found 702.2862.

Compound 1gh. R_f 0.4 (DCM/MeOH = 6/1); $[\alpha]_D^{29}$ = +20.83 (c = 3.31, MeOH/DCM = 1/1); ^1H NMR (400 MHz, MeOD/$CDCl_3$ = 1/1) δ 1.31-1.66 (m, 16H), 2.21 (t, *J* = 7.6 Hz, 2H, H-1”), 2.59 (t, *J* = 7.6 Hz, 2H), 2.66 (t, *J* = 7.6 Hz, 2H, H-10”), 3.51-3.83 (m, 8H), 3.87 (dd, *J* = 4.6, 10.6 Hz, 1H), 3.88 (d, *J* = 2.2 Hz, 1H), 4.20 (td, *J* = 4.5, 5.8 Hz, 1H), 4.88 (1H merged with water peak), 7.12-7.15 (m, 3H), 7.21 (d, *J* = 7.2 Hz, 1H), 7.23 (d, *J* = 7.4, 1H), 7.31 (d, *J* = 7.8 Hz, 2H), 7.50 (d, *J* = 8.0 Hz, 2H). ^{13}C NMR (100 MHz, MeOD/$CDCl_3$ = 1/1) δ 26.68, 27.71, 29.78, 30.13, 30.40, 32.16, 32.25, 32.91, 36.54, 36.56, 37.05, 51.56, 58.68, 68.03, 69.98, 70.78, 71.29, 72.29, 72.68, 75.39, 100.88, 125.94, 126.50, 129.09 (2C), 129.20 (4C), 129.76 (2C), 143.48, 148.34, 175.53. HRMS (FAB) *m/z* calcd for $C_{35}H_{50}NO_9F_3Na$ $[M+Na]^+$ 708.3335, found 708.3340.

Compound 1gi. R_f 0.5 (DCM/MeOH = 6/1); $[\alpha]_D^{29}$ = +19.12 (c = 5.25, MeOH/DCM = 1/1); ^1H NMR (400 MHz, MeOD): δ 1.30 (brs, 10H), 1.53-1.60 (m, 8H), 2.17 (t, *J* = 7.7 Hz, 2H, H-1”), 2.56 (t, *J* = 7.7 Hz, 2H), 2.63 (t, *J* = 7.7 Hz, 2H, H-10”), 3.50-3.78 (m, 8H), 3.84 (dd, *J* = 4.7, 10.7 Hz, 1H), 3.88 (d, *J* = 3.0 Hz, 1H), 4.15 (m, 1H), 4.86 (d, *J* = 3.8 Hz, 1H), 7.12-7.14 (m, 3H), 7.20-7.22 (m, 2H), 7.26 (d, *J* = 8.0 Hz, 2H), 7.47 (d, *J* = 8.1 Hz, 2H). ^{13}C NMR (100 MHz, MeOD): δ 26.89, 27.06, 30.20, 30.34 (3C), 30.56, 32.37, 32.74, 32.89, 36.67, 36.90, 37.21, 51.80, 62.75, 68.23, 70.22, 71.04, 71.51, 72.62, 72.85, 75.50, 101.14, 126.10, 126.13, 126.62, 129.24 (2C), 129.3 (4C), 130.02 (2C), 143.92, 148.73, 175.82; HRMS (FAB) m/z calcd for $C_{37}H_{54}NO_9F_3Na$ $[M+Na]^+$ 736.3648, found 736.3648.

Compound 1ha. R_f 0.4 (DCM/MeOH = 6/1); $[\alpha]_D^{30}$ = +40.55 (c = 2.2, MeOH/DCM = 1/1); ^1H NMR (600 MHz, MeOD): δ 0.90-1.69 (m, 38H), 2.26 (t, *J* = 7.4 Hz, 2H, H-1”), 3.58-3.86 (m, 8H), 3.92 (m, 2H), 4.24 (td, *J* = 5.1, 9.2 Hz, 1H), 4.87 (1H merge with water); ^{13}C NMR

(150 MHz, MeOD): δ 14.55, 23.14, 23.23, 23.83, 27.22, 27.50, 28.45, 29.28, 30.34, 30.52, 30.85, 33.03, 33.20, 34.14, 37.41, 38.46, 38.60, 38.64, 40.70, 55.99, 62.94, 68.50, 70.38, 71.27, 71.68, 72.66, 73.14, 75.66, 101.28, 176.01; HRMS (ESI-TOF) m/z calcd for $C_{30}H_{59}NO_9Na$ $[M+Na]^+$ 600.4082, found 600.4078.

Compound 1hb. R_f 0.4 (DCM/MeOH = 6/1); $[\alpha]_D^{29}$ = +4.21 (c = 2.6, MeOH/DCM = 1/1); ^1H NMR (600 MHz, MeOD/$CDCl_3$ = 1/1): δ 0.83-1.60 (m, 74H), 2.20 (t, *J* = 7.5 Hz, 2H, H-1"), 3.53-3.82 (m, 8H), 3.86 (dd, *J* = 4.5, 10.6 Hz, 1H), 3.89 (d, *J* = 3.0 Hz, 1H), 4.18 (td, *J* = 4.8, 10.0 Hz, 1H), 4.87 (d, *J* = 3.6 Hz, 1H); ^{13}C NMR (100 MHz, MeOD/$CDCl_3$ = 1/1): δ 14.55, 20.21, 23.12, 23.21, 23.56, 25.76, 26.89, 27.20, 28.18, 28.93, 30.27 (2C), 30.38, 30.49, 30.57 (16C), 32.86, 33.05, 33.79, 37.23, 38.16, 38.29, 40.35, 51.54, 62.62, 68.12, 69.96, 70.77, 71.26, 72.12, 72.84, 75.30, 100.81, 175.62; HRMS (ESI-TOF) m/z calcd for $C_{48}H_{95}NO_9Na$ $[M+Na]^+$ 852.6899, found 852.6893.

Compound 1hc. R_f 0.4 (DCM/MeOH = 6/1); $[\alpha]_D^{29}$ = +10.94 (c = 1.6, MeOH/DCM = 1/1); ^1H NMR (400 MHz, MeOD): δ 0.82 (d, *J* = 6.60 Hz, 3H), 0.88 (d, *J* = 6.7 Hz, 6H), 1.00-1.72 (m, 16H), 3.61 (ddd, *J* = 2.5, 4.6, 9.1 Hz, 1H), 3.65-3.86 (m, 7H), 3.87 (dd, *J* = 0.7, 3.3 Hz, 1H), 4.01 (dd, *J* = 4.4, 10.8 Hz, 1H), 4.44 (td, *J* = 5.0, 10.8 Hz, 1H), 4.92 (d, *J* = 3.7 Hz, 1H), 7.47-7.84 (m, 4H); ^{13}C NMR (100 MHz, MeOD): δ 20.23, 23.16, 23.25, 26.06, 27.35, 28.28, 29.30, 33.30, 34.12, 38.43, 38.57, 40.68, 52.98, 62.94, 68.20, 70.35, 71.21, 71.64, 72.81, 73.00, 75.52, 101.36, 129.85 (2C), 130.36 (2C), 134.59, 138.88, 168.85; HRMS (ESI-TOF) *m/z* calcd for $C_{29}H_{48}ClNO_9Na$ $[M+Na]^+$ 612.2910, found 612.2906.

Compound 1hd. R_f 0.4 (DCM/MeOH = 6/1); $[\alpha]_D^{28}$ = +38.44 (c = 1.5, MeOH/DCM = 1/1); ^1H NMR (400 MHz, MeOD): δ 0.85 (d, *J* = 6.5 Hz, 3H), 0.86 (d, *J* = 6.6 Hz, 6H), 1.05-1.63 (m, 16H), 2.57 (dt, *J* = 2.4, 7.4 Hz, 2H, H-1"), 3.12 (t, *J* = 7.4 Hz, 2H, H-2"), 3.46-3.80 (m, 8H), 3.83 (dd, *J* = 4.2, 10.5 Hz, 1H), 3.85 (d, *J* = 4.1 Hz, 1H), 4.19 (td, *J* = 4.7, 10.5 Hz, 1H), 4.83 (d, *J* = 3.7 Hz, 1H), 6.82-7.60 (m, 3H); ^{13}C NMR (100 MHz, MeOD): δ 20.30, 23.25,

26.09, 26.40, 26.93, 27.45, 28.48, 29.31, 33.40, 34.15, 38.49, 38.65, 39.17, 40.71, 52.03, 62.91, 68.30, 70.35, 71.19, 71.62, 72.73, 73.01, 75.74, 101.30, 124.54, 125.86, 127.95, 144.72, 174.31; HRMS (ESI-TOF) *m/z* calcd for $C_{29}H_{51}NO_9SNa$ [M+Na]$^+$ 612.3177, found 612.3174.

Compound 1he. R*f* 0.4 (DCM/MeOH = 6/1); $[\alpha]_D^{30}$ = +11.73 (c = 1.53, MeOH/DCM = 1/1); ^{1}H NMR (400 MHz, MeOD): δ 0.87 (d, *J* = 6.5 Hz, 3H), 0.88 (d, *J* = 6.6 Hz, 6H), 1.10-1.64 (m, 19H), 2.50 (t, *J* = 7.6 Hz, 2H, H-1”), 2.86 (t, *J* = 7.6 Hz, 2H, H-2”), 3.52 (m, 2H), 3.64 (dd, *J* = 4.7, 10.7 Hz, 1H), 3.67-3.79 (m, 5H), 3.76 (s, 3H, OMe), 3.83 (dd, *J* = 4.6, 10.7 Hz, 1H), 3.87 (d, *J* = 2.5 Hz, 1H), 4.20 (td, *J* = 4.7, 11.0 Hz, 1H), 4.85 (d, *J* = 3.8 Hz, 1H), 6.83-7.13 (m, 4H); ^{13}C NMR (100 MHz, MeOD): δ 20.29, 23.16, 23.25, 26.08, 27.41, 28.45, 29.30, 32.19, 33.40, 34.16, 38.45, 38.61, 39.37, 40.71, 51.99, 55.82, 62.94, 68.30, 70.36, 71.21, 71.64, 72.75, 73.03, 75.78, 101.29, 115.05 (2C), 130.47 (2C), 134.30, 159.73, 175.08; HRMS (ESI-TOF) *m/z* calcd for $C_{32}H_{55}NO_{10}Na$ [M+Na]$^+$ 636.3718, found 636.3720.

Compound 1hf. R*f* 0.4 (DCM/MeOH = 6/1); $[\alpha]_D^{30}$ = +32.29 (c = 1.77, MeOH/DCM = 1/1); ^{1}H NMR (400 MHz, MeOD): δ 0.87 (d, *J* = 6.6 Hz, 3H), 0.88 (d, *J* = 6.6 Hz, 6H), 1.12-1.64 (m, 16H), 2.52 (t, *J* = 7.7 Hz, 2H, H-1”), 2.91 (t, *J* = 7.7 Hz, 2H, H-2”), 3.47-3.80 (m, 8H), 3.84 (dd, *J* = 4.5, 10.7 Hz, 1H), 3.88 (d, *J* = 2.5 Hz, 1H), 4.2 (td, *J* = 4.8, 10.2 Hz, 1H), 4.91 (merged with water peak, 1H), 6.99-7.23 (m, 4H); ^{13}C NMR (100 MHz, MeOD): δ 20.28, 23.15, 23.24, 26.07, 27.41, 28.45, 29.30, 32.14, 33.45, 34.15, 38.47, 38.61, 39.07, 40.70, 51.99, 62.93, 68.30, 70.34, 71.21, 71.64, 72.76, 73.04, 75.78, 101.28, 116.08, 116.29, 131.23 138.31, 161.81, 164.22, 174.76; HRMS (ESI-TOF) *m/z* calcd for $C_{31}H_{52}FNO_9Na$ [M+Na]$^+$ 624.3518, found 624.3514.

Compound 1hg. R*f* 0.4 (DCM/MeOH = 6/1); $[\alpha]_D^{31}$ = +1.85 (c = 1.37, MeOH/DCM = 1/1); ^{1}H NMR (400 MHz, MeOD): δ 0.82 (d, *J* = 6.5 Hz, 3H), 0.88 (s, *J* = 6.6 Hz, 6H), 0.99-1.59 (m, 16H), 3.49 (m, 1H), 3.56 (dd, *J* = 2.5, 8.5 Hz, 1H, H-1’-a), 3.59-3.77 (m, 7H), 3.72 (s, 2H), 3.87 (dd, *J* = 4.1, 10.6 Hz, 1H), 4.2 (td, *J* = 4.4, 10.6 Hz, 1H), 4.84 (d, *J* = 3.9 Hz, 1H), 7.44-

7.84 (m, 7H); ^{13}C NMR (100 MHz, MeOD): δ 20.23, 23.16, 23.26, 26.10, 27.44, 28.44, 29.31, 33.17, 34.08, 38.39, 38.64, 40.72, 44.39, 52.21, 62.99, 68.44, 70.34, 71.17, 71.60, 72.70, 73.20, 75.51, 101.36, 126.98, 127.42, 128.45, 128.85, 128.89, 129.03, 129.49, 134.08, 134.59, 135.19, 173.60; HRMS (ESI-TOF) *m/z* calcd for $C_{34}H_{53}NO_9Na$ $[M+Na]^+$ 642.3613, found 642.3607.

Compound 1hh. R_f 0.4 (DCM/MeOH = 6/1); $[\alpha]_D^{28}$ = +6.25 (c = 1.43, MeOH/DCM = 1/1); ^{1}H NMR (400 MHz, MeOD): δ 0.86 (d, *J* = 6.0 Hz, 3H), 0.87 (s, *J* = 6.6 Hz, 6H), 1.10-1.69 (m, 22H), 2.22 (t, *J* = 7.5 Hz, 2H), 2.61 (t, *J* = 7.7 Hz, 2H), 3.55-3.72 (m, 5H), 3.74 (dd, *J* = 3.0, 7.1 Hz, 1H), 3.79 (dd, *J* = 3.6, 10.1 Hz, 1H), 3.83 (t, *J* = 6.1 Hz, 1H), 3.88 (m, 2H), 4.21 (td, *J* = 4.7, 10.3 Hz, 1H), 4.90 (1H merged with water), 7.11-7.26 (m, 5H); ^{13}C NMR (100 MHz, MeOD): δ 18.72, 21.60, 21.69, 24.51, 25.50, 25.99, 26.90, 27.73, 28.55, 29.33, 31.07, 32.59, 35.33, 35.76, 36.94, 37.09, 39.14, 50.43, 61.34, 66.86, 68.82, 69.64, 70.11, 71.18, 71.55, 74.11, 99.73, 125.26, 127.86 (2C), 127.98 (2C), 142.35, 174.37; HRMS (ESI-TOF) *m/z* calcd for $C_{34}H_{59}NO_9Na$ $[M+Na]^+$ 648.4082, found 648.4085.

Compound 1hi. R_f 0.4 (DCM/MeOH = 6/1); $[\alpha]_D^{29}$ = +35.99 (c = 1.57, MeOH/DCM = 1/1); ^{1}H NMR (400 MHz, MeOD): δ 0.86 (d, *J* = 6.5 Hz, 3H), 0.87 (s, *J* = 6.6 Hz, 6H), 1.07-1.63 (m, 26H), 2.22 (t, *J* = 7.5 Hz, 2H), 2.60 (t, *J* = 7.7 Hz, 2H), 3.55 (m, 1H), 3.61 (t, *J* = 6.0 Hz, 1H), 3.65-3.80 (m, 5H), 3.83 (t, *J* = 6.0 Hz, 1H), 3.88 (m, 2H), 4.20 (td, *J* = 4.5, 10.4 Hz, 1H), 4.90 (1H merged with water), 7.11-7.25 (m, 5H); ^{13}C NMR (100 MHz, MeOD): δ 20.29, 23.16, 23.25, 26.09, 27.21, 27.55, 28.45, 29.30, 30.37, 30.51, 30.89, 32.91, 33.17, 34.16, 37.07, 37.38, 38.50, 38.63, 40.71, 51.99, 62.91, 68.44, 70.38, 71.21, 71.66, 72.75, 73.10, 75.61 101.29, 126.77, 129.39 (2C), 129.52 (2C), 144.07, 175.98; HRMS (ESI-TOF) *m/z* calcd for $C_{36}H_{63}NO_9Na$ $[M+Na]^+$ 676.4395, found 676.4388.

Compound 6a. To a solution of **11** (1.5 g, 3.26 mmol) and triphenyl phosphine (1.0 g, 3.92 mmol) in THF (30.0 mL) was added diisopropyl azodicaboxylate (0.8 mL, 3.92 mmol) at 0 ^{o}C. Subsequently, 2-mercaptobenzothiazole (655.6 mg, 3.92 mmol) was added and the resulting

solution was stirred under N_2 while gradually warmed to rt. After 5 h, TLC showed complete conversion and the reaction was quenched with brine. The aqueous layer was extracted with EtOAc (x2) and the combined organic layers were dried ($MgSO_4$) and concentrated. Purification by column chromatography gave **6a** (1.7 g, 84%) as a yellow oil, R_f 0.55 (n-hexanes/EtOAc = 3/1); $[\alpha]_D^{31}$ = +2.63 (c = 3.3, CH_2Cl_2): ^{1}H NMR (400 MHz, $CDCl_3$): δ 0.88 (s, 3H), 1.12 (s, 3H), 3.29 (ddd, *J* = 2.7, 6.4, 9.5 Hz, 1H), 3.41 (dd, *J* = 6.4, 10.0 Hz, 1H), 3.51 (dd, *J* = 2.7, 10.0 Hz, 1H), 3.73 (dd, *J* = 2.4, 14.4 Hz, 1H),3.82 (dt, *J* = 10.3, 14.4 Hz, 1H), 4.12 (dd, *J* = 5.7, 9.5 Hz, 1H), 4.75 (dt, *J* = 2.4, 5.7, 10.3 Hz, 1H), 7.15-8.10 (m, 19H); ^{13}C NMR (100 MHz, $CDCl_3$): δ 25.47, 27.87, 33.88, 60.67, 63.64, 66.60, 76.06, 81.99, 109.52, 120.97, 121.52, 124.30, 126.03, 127.12 (3C), 127.83 (6C), 128.56 (6C), 135.27, 143.79 (3C), 152.95, 166.10; HRMS (ESI-TOF) *m/z* calcd for $C_{34}H_{32}N_4O_3S_2Na$ $[M+Na]^+$ 631.1808, found 631.1809.

Compound 9. To a stirred solution of **8**[2] (10 g, 23.1 mmol) in MeOH (200 mL) at 0 °C was added sodium borohydride (1.3 g, 34.7 mmol) dropwise. After being stirred at 0 °C for 3 h, the reaction mixture was quenched by adding AcOH and the resulting solution was concentrated. The residue was diluted with EtOAc and the resulting organic layer was washed by H_2O and brine. The organic layer was dried over anhydrous $MgSO_4$ and then concentrated. The crude product was purified by flash chromatography to obtain a colorless oil **9** (9.5 g, 94%), R_f 0.2 (n-hexanes/EtOAc = 2/1); $[\alpha]_D^{28}$ = +9.58 (c = 8, CH_2Cl_2); ^{1}H NMR (400 MHz, MeOD): δ 1.33 (s, 3H), 1.45 (s, 3H), 3.25 (ddd, *J* = 6.2, 9.1, 13.0 Hz, 2H), 3.72 (d, *J* = 5.2 Hz, 2H), 3.87 (dt, *J* = 2.6, 6.2 Hz, 1H), 4.19 (dt, *J* = 5.2, 6.8 Hz, 1H), 4.37 (dd, *J* = 2.6, 6.8 Hz, 1H), 7.17-7.27 (m, 9H), 7.45-7.48 (m, 6H). ^{13}C NMR (100 MHz, MeOD): δ 25.39, 27.27, 62.01, 66.42, 69.32, 77.62, 78.93, 87.94, 109.14, 128.04 (3C), 128.72 (6C), 129.77 (6C), 145.24 (3C). HRMS (ESI) *m/z* calcd for $C_{27}H_{30}O_5Na$ $[M+Na]^+$ 457.1944, found: 457.1991.

Compound 10. To a solution of **9** (2 g, 4.6 mmol) in 50 mL of pyridine in an ice bath was

added dropwise benzoyl chloride (0.6 mL, 5.06 mmol). The mixture was stirred at 0 °C for 3 h, and then the reaction was quenched with MeOH. After the mixture was concentrated, the residue was diluted with EtOAc and then washed with H_2O. The organic layer was dried over anhydrous $MgSO_4$ and then concentrated. The crude product was purified by flash chromatography to obtain a colorless oil **10** (2.2 g, 89%), R_f 0.45 (n-hexanes/EtOAc = 3/1); $[\alpha]_D^{30}$ = +6.78 (c = 11, CH_2Cl_2): ^{1}H NMR (400 MHz, $CDCl_3$): δ 1.37 (s, 3H), 1.48 (s, 3H), 3.23 (dd, *J* = 5.8, 9.4 Hz, 1H), 3.28 (dd, *J* = 5.8, 9.4 Hz, 1H), 3.87 (dt, *J* = 2.8, 5.8 Hz, 1H), 4.39 (dt, *J* = 2.8, 7.2 Hz, 1H), 4.46 (dd, *J* = 6.6, 13.1 Hz, 2H), 4.53 (dt, *J* = 7.2, 13.1 Hz, 1H), 7.20-8.04 (m, 20H). ^{13}C NMR (100 MHz, $CDCl_3$): δ 25.16, 27.30, 64.45, 65.34, 68.49, 75.27, 76.37, 87.09, 108.96, 125.70, 127.27 (3C), 128.02 (6C), 128.51 (2C), 128.78 (6C), 129.87 (2C), 133.21, 143.88 (3C), 166.41; HRMS (ESI-TOF) *m/z* calcd for $C_{34}H_{34}O_6Na^+$ $[M+Na]^+$ 561.2248, found 561.2249.

Compound 11. To a solution **10** (2.5 g, 4.6 mmol) and PPh_3 (1.5 g, 5.6 mmol) in THF (50 mL) was added diisopropyl azodicaboxylate (1.1 mL, 5.6 mmol) at 0 °C. Subsequently, diphenylphosphoryl azide (1.2 mL, 5.6 mmol) was added to the mixture. After the reaction was stirred for overnight, the mixture was poured into H_2O and extracted with EtOAc. The organic layer was washed with brine, dried over $MgSO_4$, and concentrated to afford a crude residue. The residue was purified by flash column chromatography on silica gel eluting with *n*-hexanes/EtOAc (3/1) to get yellow oil (2.2 g, 90%; R_f 0.25 in n-hexanes/EtOAc = 3/1). The intermediate was treated with NaOMe (65 mg, 1.2 mmol) in MeOH (40 mL). The reaction mixture was stirred at rt for 1 h and then neutralized with Amberlite IR-120 (H^+) resin. The mixture was filtrated and the MeOH was evaporated. The residue was purified by flash chromatography to afford a colorless syrup **11** (1.6 g, 87%). R_f 0.25 (n-hexanes/EtOAc = 3/1); $[\alpha]_D^{30}$ = +4.33 (c = 11, CH_2Cl_2): ^{1}H NMR (400 MHz, $CDCl_3$): δ 1.28 (s, 3H), 1.30 (s, 3H), 2.05 (s, 1H), 3.36 (dd, *J* = 7.3, 10.1 Hz, 1H), 3.55 (dd, *J* = 2.6, 10.1 Hz, 1H), 3.61 (ddd, *J* = 2.6, 7.3, 9.9 Hz, 1H), 3.72 (dd, *J* = 6.0, 11.7 Hz, 1H), 3.79 (dd, *J* = 4.8, 11.7 Hz, 1H), 3.99 (dd, *J* = 5.9,

9.9 Hz, 1H), 4.22 (dt, *J* = 4.8, 5.9 Hz, 1H), 7.20-7.47 (m, 15H); ^{13}C NMR (100 MHz, $CDCl_3$): δ 25.49, 27.96, 60.35, 61.12, 64.53, 75.06, 77.77, 87.60, 109.01, 127.34 (3C), 128.08 (6C), 143.84 (3C); HRMS (ESI-TOF) *m/z* calcd for $C_{27}H_{29}N_3O_4Na^+$ $[M+Na]^+$ 482.2050, found 482.2055.

Compound 12. Tosyl chloride (3.15 g, 16.54 mmol) was added to a solution of **11** (2.5 g, 5.51 mmol) in pyridine (55 mL) at ice bath temeprature. After 2 h, the reaction was quenched with methanol and the reaction mixture was concentrated to afford crude residues. Then, it was extracted with EtOAc (x2) and the combined organic layers were dried ($MgSO_4$) and concentrated. Purification by column chromatography gave **12** (2.95 g, 87%) as a yellow oil. R_f 0.50 (Hexanes/EtOAc = 4/1); ^1H NMR (400 MHz, CDCl3): δ 1.23 (s, 6H), 2.42 (s, 3H), 3.34 (dd, *J* = 6.0, 12.3 Hz, 1H, H-5), 3.36 (dd, *J* = 6.0, 12.3 Hz, 1H, H-5'), 3.46 (m, 1H, H-2), 4.0 (m, 2H, H-1), 4.21 (dd, *J* = 3.9, 10.3 Hz, 1H, H-3), 4.25 (dt, *J* = 3.9, 6.0 Hz, 1H, H-4), 7.15-8.10 (m, 19H); ^{13}C NMR (100 MHz, CDCl3): δ 21.87, 25.60, 27.71, 59.77, 64.28, 68.21, 74.82, 75.10, 87.63, 109.83, 127.39 (3C), 128.10 (6C), 128.28 (2C), 128.87 (6C), 130.10 (2C), 133.00, 143.72 (3C), 145.26; HRMS (ESI-TOF) calcd for $C_{34}H_{35}N_3O_6SNa^+$ $[M+Na]^+$: 636.2139, found :636.2140.

Compound 13. $P(OEt)_3$ (5 mL) and **12** (165 mg, 0.27 mmol) was combined and the solution was refluxed at 150 ^oC for 16 h. The reaction mixture was concentrated and extracted with EtOAc (x2). Then, the combined organic layers were dried ($MgSO_4$) and concentrated. Purification by column chromatography obtained **13** (114 mg, 73%) as a yellow oil. R_f 0.15 (Hexanes/EtOAc = 4/1); ^1H NMR (400 MHz, d-Acetone): δ 1.21 (t, *J* = 7.1 Hz, 3H), 1.22 (t, *J* = 7.0 Hz, 3H), 1.25 (s, 3H), 1.41 (s, 3H), 3.20 (dd, *J* = 4.3, 9.6 Hz, 1H, H-1), 3.26 (dd, *J* = 5.1, 9.6 Hz, 1H, H-1'), 3.41 (dd, *J* = 4.5, 11.6 Hz, 1H, H-5), 3.50 (ddd, *J* = 3.1, 4.5, 11.6 Hz, 1H, H-5'), 3.85 (dt, *J* = 4.3, 9.1 Hz, 1H, H-2), 3.90-4.0 (m, 4H, H-1), 4.59 (dd, *J* = 3.1, 5.8 Hz, 1H, H-4), 4.82 (m, 1H, H-3), 7.25-7.48 (m, 15H); ^{13}C NMR (100 MHz, d-Acetone): δ 16.64, 24.44, 26.61, 54.12, 62.51, 65.64, 65.83, 81.79, 81.86, 84.26, 84.34, 88.18, 111.84, 128.06 (3C),

128.80 (6C), 129.68 (6C), 144.91 (3C); HRMS (ESI-TOF) calcd for $C_{31}H_{38}N_3O_6PNa^+$ $[M+Na]^+$: 602.2390, found : 602.2395.

Compound 14. To a solution of **6a** (1.35 g, 2.22 mmol) in CH_2Cl_2 (30 mL) at 0 ℃ was added solid $NaHCO_3$ (932 mg, 11.10 mmol) and followed by addition of *m*-CPBA (1.15 g, 6.66 mmol). The resulting suspension was warmed to rt and stirred for overnight. The reaction was quenched with aq. $Na_2S_2O_3$ (sat.) and diluted with CH_2Cl_2. The organic layer was washed with aq. $Na_2S_2O_3$ solution (sat. 3x) and the combined aqueous layers were subsequently extracted with CH_2Cl_2 (3x). The combined organic layers were washed with aq. $NaHCO_3$ (sat. 3x) and brine. The organic layer was dried ($MgSO_4$) and concentrated. The product was purified by column chromatography to give **14** (1.2 g, 85%) as a colorless oil. R_f 0.3 (Hexanes/EtOAc = 3/1); $[\alpha]_D^{29}$ = +12.52 (c = 5, CH_2Cl_2): ^{1}H NMR (400 MHz, $CDCl_3$): δ 0.88 (s, 3H), 1.12 (s, 3H), 3.29 (ddd, *J* = 2.7, 6.5, 9.6 Hz, 1H), 3.41 (dd, *J* = 6.5, 10.3 Hz, 1H), 3.51 (dd, *J* = 2.7, 10.3 Hz, 1H), 3.73 (dd, *J* = 2.4, 14.4 Hz, 1H), 3.82 (dd, *J* = 10.4, 14.4 Hz, 1H), 4.12 (dd, *J* = 5.6, 9.6 Hz, 1H), 4.75 (ddd, *J* = 2.4, 5.6, 10.4 Hz, 1H), 7.22-7.63 (m, 17H), 7.98-8.02 (m, 1H), 8.18-8.21 (m, 1H). ^{13}C NMR (100 MHz, $CDCl_3$): δ 25.77, 28.21, 57.50, 59.36, 59.87, 64.28, 71.25, 75.54, 87.70, 110.46, 122.59, 124.21, 126.50, 127.25, 127.40 (3C), 128.11 (6C), 136.21, 143.67 (3C), 154.11, 178.12; HRMS (ESI-TOF) *m/z* calcd for $C_{34}H_{32}N_4O_5S_2Na$ $[M+Na]^+$ 663.1706, found 663.1710.

Compound 26. TMSI (0.4 mL, 2.96 mmol) was added to a solution of the **24**[3] (1.44 g, 2.69 mmol) in CH_2Cl_2 (27 mL) at 0 °C. The reaction mixture was stirred at 0 °C for 30 min and then 10 mL of anhydrous toluene were added. After azeotroping three times with toluene, the crude product **25** was dissolved in toluene (10 mL) and kept under N_2. In a separate flask, 4Å MS (2.0 g), TBAI (1.42 g, 3.84 mmol), **6c** (1.2 g, 2.24 mmol), and diisopropylethylamine (0.56 mL, 3.36 mmol) were mixed in toluene (20 mL). The mixture was stirred under N_2 at 90 °C for 20 min, and then **25** was cannulated into the reaction mixture. After the reaction mixture was stirred at 90 °C for 2 h, the solution was filtered and extracted with EtOAc and $Na_2S_2O_{3(aq.)}$.

The organic layer was dried with $MgSO_4$ and evaporated to dryness. The residue was purified by flash column chromatography on silica gel (n-hexanes : EtOAc 4:1) to give product **26** (0.8 g, 45%) as a colorless oil. R_f 0.4 (Hexanes/EtOAc = 4:1); $[\alpha]_D^{30}$ = +20.54 (c = 2.5, CH_2Cl_2); ^1H NMR (400 MHz, $CDCl_3$): δ 1.74-2.0 (m, 7H), 3.26 (t, *J* = 6.8 Hz, 2H, H-1), 3.52-3.75 (m, 7H, H-4, H-6, H-2', H-3', H-4', H-6'), 3.87 (m, 1H, H-5'), 3.96 (m, 2H, H-7), 4.07 (dd, *J* = 3.3, 10.0 Hz, 1H, H-5), 4.48 (d, *J* = 11.5 Hz, 1H), 4.56-4.71 (m, 5H), 4.75 (dd, *J* = 11.8 Hz, 1H), 4.87 (d, *J* = 11.8 Hz, 1H), 4.90 (d, *J* = 3.5 Hz, 1H, H-1'), 4.96 (dd, *J* = 11.6 Hz, 1H), 7.22-7.84 (m, 29H); ^{13}C NMR (100 MHz, CDCl3): δ 25.22, 28.98, 31.50, 33.70, 62.12, 62.58, 68.57, 71.13, 72.20, 73.49, 73.56, 73.95, 74.69, 75.32, 76.59, 78.75, 78.87, 79.00, 98.84, 121.14, 121.66, 124.37, 126.23, 127.78 (2C), 127.93 (2C), 128.03, 128.12 (4C), 128.26, 128.29, 128.37, 128.40, 128.49 (2C), 128.61 (6C), 128.66 (2C), 128.75 (2C), 135.36, 138.06, 138.18, 138.80, 138.87, 153.48, 167.23, 170.76; HRMS (ESI-TOF) calcd for $C_{57}H_{60}N_4O_9S_2Na^+$ $[M+Na]^+$: 1031.3694; Found: 1031.3698.

Compound 34. Yield (210mg, 90%). R_f 0.5 (n-hexanes/EtOAc = 4/1); $[\alpha]_D^{28}$ = +24.55 (c = 2.53, CH_2Cl_2); ^1H NMR (400 MHz, $CDCl_3$): δ 0.89 (t, *J* = 6.8 Hz, 3H), 1.27-2.28 (m, 26H), 3.58 (m, 1H), 3.67-3.77 (m, 4H), 3.88 (dt, *J* = 1.8, 12.6 Hz, 1H), 4.01 (dd, *J* = 3.1, 6.2 Hz, 1H), 4.05 (t, *J* = 5.8 Hz, 2H, $F_{tag}CH_2CH_2C\mathit{H}_2O$), 4.08-4.18 (m, 2H), 4.09 (dd, *J* = 3.3, 10.0 Hz, 1H), 4.16 (d, *J* = 3.0 Hz, 1H), 4.50 (dd, *J* = 2.9, 11.5 Hz, 1H), 4.58-4.70 (m, 4H), 4.74 (d, *J* = 12.3 Hz, 1H), 4.81 (d, *J* = 12.3 Hz, 1H), 4.86 (d, *J* = 11.9 Hz, 1H), 4.98 (d, *J* = 3.3 Hz, 1H), 5.38 (m, 2H), 5.42 (s, 1H), 6.88 (d, *J* = 8.8 Hz, 2H), 7.23-7.34 (m, 18H), 7.40 (d, *J* = 6.9 Hz, 2H), 7.45 (d, *J* = 8.7 Hz, 2H); ^{13}C NMR (100 MHz, $CDCl_3$): δ 14.29, 20.84, 22.89, 27.60, 28.24, 28.74, 29.56, 29.61, 29.87 (3C), 30.32, 32.14, 32.84, 62.05, 63.26, 66.66, 68.75, 69.53, 72.28, 72.35, 73.74, 74.02, 74.91, 75.75, 76.11, 79.16, 79.30, 99.43, 101.15, 114.34 (2C), 127.69, 127.74, 127.85, 127.92 (4C), 127.97 (4C), 128.04 (2C), 128.46 (2C), 128.49 (2C), 128.60 (3C), 129.18, 129.65, 130.99, 131.23, 131.52, 138.31, 138.63, 139.03 (2C), 159.27; HRMS (ESI-

TOF) *m/z* calcd for $C_{70}H_{78}F_{17}N_3O_9Na$ $[M+Na]^+$ 1450.5359, found 1450.5345.

Compound 35. Yield (666 mg, 78%). R_f 0.5 (n-hexanes/EtOAc = 4/1); $[\alpha]_D^{28}$ = +11.28(c = 3.50, CH_2Cl_2); ^{1}H NMR (400 MHz, $CDCl_3$): δ 0.87 (t, *J* = 6.8 Hz, 3H), 1.28 (m, 31H), 1.75 (m, 1H), 1.92-2.16 (m, 6H), 2.30 (m, 2H), 3.55 (m, 1H), 3.63-3.74 (m, 4H), 3.86 (dt, *J* = 1.9, 12.5 Hz, 1H), 3.98 (dd, *J* = 3.2, 6.1 Hz, 1H), 4.03 (t, *J* = 6.0 Hz, 2H, $F_{tag}CH_2CH_2CH_2O$), 4.03 (m, 1H), 4.07 (dd, *J* = 3.2, 10.0 Hz, 1H), 4.13 (d, *J* = 3.0 Hz, 1H), 4.47 (dd, *J* = 2.8, 11.5 Hz, 1H), 4.56-4.60 (m, 2H), 4.65 (d, *J* = 11.9 Hz, 1H), 4.66 (d, *J* = 11.2 Hz,1H), 4.72 (d, *J* = 12.3 Hz, 1H), 4.79 (d, *J* = 12.3 Hz, 1H), 4.84 (d, *J* = 11.9 Hz, 1H), 4.95 (d, *J* = 3.2 Hz, 1H), 5.35 (m, 2H, H-7. H-8), 5.39 (s, 1H), 6.86 (d, *J* = 8.8 Hz, 2H), 7.21-7.33 (m, 18H), 7.38 (d, *J* = 6.8 Hz, 2H), 7.42 (d, *J* = 8.7 Hz, 2H); ^{13}C NMR (100 MHz, $CDCl_3$): δ 14.15, 20.59, 22.76, 23.26, 27.45, 28.00, 28.56, 29.33, 29.45, 29.79 (8C), 30.06, 30.22, 32.01, 32.71, 61.75, 63.07, 66.37, 68.52, 69.34, 72.09, 72.19, 73.60, 73.86, 74.70, 75.53, 75.88, 78.87, 79.14, 99.23, 100.99, 114.10 (2C), 127.54, 127.60, 127.70, 127.78 (4C), 127.8 (4C), 127.92 (2C), 128.31 (2C), 128.34 (4C), 132.08, 129.01. 129.48, 130.81, 130.97, 131.37, 138.12, 138.43, 138.83 (2C), 159.10; HRMS (ESI-TOF) *m/z* calcd for $C_{77}H_{92}F_{17}N_3O_9Na$ $[M+Na]^+$ 1548.6460, found 1548.6462.

Compound 36. Yield (530 mg, 67%). R_f 0.3 (n-hexanes/EtOAc = 4/1); $[\alpha]_D^{31}$ = +21.33 (c = 5.30, CH_2Cl_2); ^{1}H NMR (400 MHz, $CDCl_3$): δ 1.67-2.37 (m, 8H), 3.57(m, 1H), 3.63-3.75 (m, 3H), 3.80 (dd, *J* = 3.5, 6.8 Hz, 1H), 3.89 (dd, *J* = 1.5, 12.6 Hz, 1H), 4.00-4.11 (m, 4H), 4.04 (t, *J* = 5.8 Hz, 2H, $F_{tag}CH_2CH_2CH_2O$), 4.15 (d, *J* = 2.8 Hz, 1H), 4.48 (d, *J* = 11.5 Hz, 1H), 4.61 (d, *J* = 11.1 Hz, 1H), 4.63 (d, *J* = 11.5 Hz, 1H), 4.67 (d, *J* = 11.6 Hz, 1H), 4.69 (d, *J* = 11.1 Hz, 1H), 4.74 (d, *J* = 12.3 Hz, 1H), 4.80 (d, *J* = 12.3 Hz, 1H), 4.87 (d, *J* = 11.6 Hz, 1H), 4.98 (d, *J* = 3.3 Hz, 1H), 5.41 (s, 1H), 6.11 (dt, *J* = 6.8, 15.7 Hz, 1H), 6.25 (d, *J* = 15.7 Hz, 1H), 6.88 (d, *J* = 8.7 Hz, 2H), 7.19-7.34 (m, 22H), 7.39 (d, *J* = 7.7 Hz, 2H), 7.44 (d, *J* = 8.7 Hz, 2H); ^{13}C NMR (100 MHz, $CDCl_3$): δ 20.69, 28.09, 28.32, 29.11, 61.78, 63.19, 66.49, 68.54, 69.44, 72.15, 72.18, 73.76, 74.06, 74.74, 75.60, 75.99, 78.58, 78.93, 99.35, 101.10, 114.22 (2C), 127.31,

127.68, 127.72, 127.91 (8C), 128.06 (2C), 128.10 (2C), 128.42 (2C), 128.46 (2C), 128.59 (4C), 128.76 (2C), 129.27, 130.23, 131.03, 131.17, 132.61, 136.33, 138.16, 138.37, 138.89 (2C), 159.20; HRMS (ESI-TOF) *m/z* calcd for $C_{66}H_{61}F_{17}N_3O_9Na$ $[M+Na]^+$ 1420.3723, found1420.3729.

Compound 37. Yield (600 mg, 93%). R_f 0.6 (n-hexanes/EtOAc = 4/1); $[\alpha]_D^{28}$ = +23.31 (c = 2.51, CH_2Cl_2); ^{1}H NMR (400 MHz, $CDCl_3$): δ 1.53 (m, 1H), 1.70 (m, 1H), 1.97-2.36 (m, 6H), 2.63 (d, *J* = 7.8 Hz, 2H, H-10), 3.54 (m, 1H), 3.61-3.70 (m, 4H), 3.85 (dd, *J* = 6.0, 11.1 Hz, 1H), 3.96-4.08 (m, 4H), 4.02 (t, *J* = 6.0 Hz, $F_{tag}CH_2CH_2C\mathit{H}_2O$), 4.12 (d, *J* = 3.0 Hz, 1H), 4.44 (d, *J* = 11.5 Hz, 1H), 4.56 (*J* = 11.6 Hz, 2H), 4.64 (d, *J* = 11.6 Hz, 1H), 4.66 (d, *J* = 7.2 Hz, 1H), 4.71 (d, *J* = 12.3 Hz, 1H), 4.78 (d, *J* = 12.3 Hz, 1H), 4.83 (d, *J* = 11.9 Hz, 1H), 4.95 (d, *J* = 2.9 Hz), 5.31-5.44 (m, 2H), 5.39 (s, 1H), 6.85 (d, *J* = 8.8 Hz, 2H), 7.12-7.31 (m, 23H), 7.37 (d, *J* = 7.8 Hz, 2H), 7.41 (d, *J* = 8.6 Hz, 2H); ^{13}C NMR (100 MHz, $CDCl_3$): δ 20.59, 23.31, 27.99, 28.50, 29.26, 36.09, 61.78, 63.06, 66.39, 68.53, 69.32, 72.07, 72.19, 73.58, 73.85, 74.68, 75.53, 75.88, 78.85, 79.15, 99.23. 100.97, 114.11 (2C), 125.84, 127.53, 127.58, 127.70, 127.75 (4C), 127.80 (4C), 127.89, 127.91, 128.29, 128.31, 128.33 (2C), 128.44 (4C), 128.48, 128.50, 129.45, 129.91, 130.21, 130.33, 130.98, 138.10, 138.37, 138.39, 138.82 (2C), 142.09, 159.09; HRMS (ESI-TOF) *m/z* calcd for $C_{68}H_{66}F_{17}N_3O_9Na$ $[M+Na]^+$ 1414.4425, found 1414.4435.

Compound 38. Yield (670 mg, 88%). R_f 0.6 (n-hexanes/EtOAc = 4/1); $[\alpha]_D^{28}$ = +28.41 (c = 1.0, CH_2Cl_2); ^{1}H NMR (400 MHz, $CDCl_3$): δ 1.24-2.20 (m, 16H), 2.29 (dd, *J* = 8.28, 18.04 Hz, 1H), 2.36 (dd, *J* = 7.64, 18.04 Hz, 1H), 2.57 (dt, *J* = 6.28, 15.16 Hz, 2H, H-13), 3.54 (m, 1H), 3.61-3.70 (m, 3H), 3.73 (dd, *J* = 3.48, 6.48 Hz, 1H), 3.86 (m, 1H), 3.95-4.06 (m, 3H), 4.02 (t, *J* = 5.94 Hz, 2H, $F_{tag}CH_2CH_2C\mathit{H}_2O$), 4.06 (dd, *J* = 3.28, 13.52 Hz, 1H), 4.12 (d, *J* = 3.00 Hz, 1H), 4.46 (d, *J* = 11.52 Hz, 1H), 4.55-4.60 (m, 1H), 4.58 (d, *J* = 11.52 Hz, 1H), 4.63-4.67 (m, 2H), 4.71 (d, *J* = 12.32 Hz, 1H), 4.78 (d, *J* = 12.32 Hz, 1H), 4.83 (d, *J* = 11.88 Hz, 1H), 4.95

(d, J = 3.28 Hz, 1H), 5.26-5.38 (m, 2H), 5.39 (s, 1H), 6.85 (d, J = 8.80 Hz, 2H), 7.13-7.31 (m, 23H), 7.36-7.43 (m, 4H); ^{13}C NMR (100 MHz, $CDCl_3$): δ 20.78, 23.45,28.19, 29.05, 29.23, 29.64, 29.78, 31.56, 31.61, 32.70, 36.13, 61.96, 63.22, 66.59, 68.71, 69.49, 72.25, 72.35, 73.73, 74.01, 74.85, 75.70, 76.06, 79.10, 79.33, 99.40, 101.13, 114.29 (2C), 125.79, 127.68, 127.73, 127.84 (2C), 127.91 (4C), 127.95 (4C), 128.03 (2C), 128.07 (2C), 128.44 (2C), 128.48 (2C), 128.58 (4C), 129.30, 129.79, 130.75, 131.16, 131.29, 138.28, 138.58, 138.99 (2C), 143.03, 159.24; HRMS (ESI-TOF) *m/z* calcd for $C_{71}H_{72}F_{17}N_3O_9Na$ $[M+Na]^+$ 1456.4889, found 1456.4894.

Compound 39. Yield (550 mg, 74%). R_f 0.6 (n-hexanes/EtOAc = 4/1); $[\alpha]_D^{28}$ = +14.57 (c = 2.0, CH_2Cl_2); ^{1}H NMR (400 MHz, $CDCl_3$): δ 0.86 (ddd, J = 1.0, 6.7, 10.4 Hz, 3H), 1.10-1.26 (m, 3H), 1.50 (d, J = 7.7 Hz, 3H), 1.59 (d, J = 8.2 Hz, 3H), 1.69-2.25 (m, 9H), 3.49 (m, 1H), 3.55-3.68 (m, 4H), 3.79 (dd, J = 4.3, 12.1 Hz, 1H), 3.90-3.97 (m, 3H), 3.95 (t, J = 5.8 Hz, 2H, $F_{tag}CH_2CH_2CH_2O$), 4.01 (dd, J = 3.3, 10.0 Hz, 1H), 4.07 (d, J = 2.9 Hz, 1H), 4.42 (dd, J = 7.3, 11.6 Hz, 1H), 4.52 (m, 2H), 4.57-4.61 (m, 2H), 4.66 (d, J = 12.3, 1H), 4.73 (d, J = 12.3, 1H), 4.78 (d, J = 11.9), 4.89 (d, J = 3.2 Hz, 1H), 5.01 (m, 1H), 5.05-5.27 (m, 2H), 5.33 (s, 1H), 6.79 (d, J = 8.7 Hz, 2H), 7.13-7.25 (m, 18H), 7.31 (d, J = 7.3 Hz, 2H), 7.36 (d, J = 8.6 Hz, 2H); ^{13}C NMR (125 MHz, $CDCl_3$): δ 17.92, 20.80, 25.92, 26.29, 28.02, 28.20, 28.38, 29.91, 31.80, 36.51, 37.42, 37.90, 51.55, 63.31, 66.61, 68.04, 69.54, 71.92, 72.50, 73.57, 73.74, 74.25, 74.54, 76.29, 79.15, 80.31, 99.70, 101.10, 114.29 (2C), 127.75 (2C), 127.82, 127.87, 127.94 (2C), 127.98 (2C), 128.0 (2C), 128.16 (2C), 128.52 (2C), 128.63 (4C), 128.69 (2C), 128.87 (2C), 131.10, 131.36, 132.96, 137.10, 137.33, 137.76, 138.49, 138.58, 138.76, 159.23; HRMS (ESI-TOF) *m/z* calcd for $C_{68}H_{72}F_{17}N_3O_9Na$ $[M+Na]^+$ 1420.4895, found 1420.4906.

Compound 40. Yield (1.0 g, 75%). R_f 0.6 (n-hexanes/EtOAc = 4/1); $[\alpha]_D^{30}$ = +23.57 (c = 5.5, CH_2Cl_2); ^{1}H NMR (400 MHz, $CDCl_3$): δ 1.54-2.36 (m, 10H), 2.64-2.70 (m, 2H, H-10), 3.55 (brs, 1H), 3.60-3.73 (m, 4H), 3.86 (d, J = 12.0 Hz, 1H), 3.95-4.05 (m, 3H), 4.02 (t, J = 6.0 Hz,

2H, $F_{tag}CH_2CH_2CH_2O$) 4.05-4.07 (m, 1H), 4.13 (brs, 1H), 4.43 (dd, *J* = 3.4, 11.4 Hz, 1H), 4.57 (d, *J* = 11.2 Hz, 1H), 4.58 (d, *J* = 11.6 Hz, 1H), 4.64 (d, *J* = 11.1 Hz, 1H), 4.66 (d, *J* = 11.2 Hz, 1H), 4.71 (d, *J* = 12.4 Hz, 1H), 4.78 (d, *J* = 12.1 Hz, 1H), 4.82 (d, *J* = 11.7 Hz, 1H), 4.94 (d, *J* = 3.3, 1H), 5.35-5.36 (m, 2H), 5.39 (s, 1H), 6.85 (m, 2H), 7.20-7.50 (m, 26H). ^{13}C NMR (100 MHz, $CDCl_3$) δ 20.57, 23.26, 27.97, 28.48, 28.81, 29.85, 34.01, 35.75, 61.72, 63.04, 66.37, 68.49, 69.31, 72.06, 73.58, 73.87, 74.64, 75.46, 75.86, 78.60, 78.86, 79.10, 99.02, 100.96, 114.08 (2C), 125.19, 127.51, 127.56, 127.71 (2C), 127.73 (4C), 127.78 (2C), 127.80 (2C), 127.83 (2C), 127.87 (2C), 128.30 (2C), 128.31 (2C), 128.42 (4C), 128.77, 129.44, 130.39, 130.90, 138.05, 138.30, 138.78 (2C), 146.19, 159.06. HRMS (FAB) *m/z* calcd for $C_{69}H_{65}N_3O_9F_{20}Na$ $[M+Na]^+$ 1482.4299, found 1482.4297.

Compound 41. Yield (132.4 mg, 38%). R_f 0.4 (n-hexanes/EtOAc = 4/1); $[\alpha]_D^{31}$ = +19.13 (c = 2.11, CH_2Cl_2); ^1H NMR (400 MHz, $CDCl_3$): δ 1.58-2.39 (m, 8H), 3.52 (dd, *J* = 6.5, 13.7 Hz, 2H), 3.57(m, 1H), 3.64-3.80 (m, 4H), 3.88 (m, 1H), 4.0-4.11 (m, 4H), 4.04 (t, *J* = 5.9 Hz, 2H, $F_{tag}CH_2CH_2CH_2O$), 4.15 (d, *J* = 2.5 Hz, 1H), 4.49 (t, *J* = 10.5 Hz, 1H), 4.58-4.69 (m, 4H), 4.74 (d, *J* = 12.3 Hz, 1H), 4.81 (d, *J* = 12.4 Hz, 1H), 4.86 (d, *J* = 11.8 Hz, 1H), 4.98 (d, *J* = 3.0 Hz, 1H), 5.42 (s, 1H), 5.46-5.67 (m, 2H), 6.76-7.34 (m, 23H), 7.40 (d, *J* = 7.0 Hz, 2H), 7.44 (d, *J* = 8.6 Hz, 2H); ^{13}C NMR (100 MHz, $CDCl_3$): δ 20.72, 24.20, 25.87 27.90, 28.12, 28.34, 29.88, 61.81, 63.17, 66.52, 68.65, 69.46, 72.24, 73.73, 73.95, 74.79, 75.63, 75.97, 78.83, 79.16, 79.27, 99.33, 101.11, 114.23 (2C), 123.63, 124.22, 127.01, 127.73, 127.90 (8C), 127.93 (6C), 128.43 (2C), 128.47 (2C), 128.57 (4C), 129.05, 131.06, 138.16, 138.26, 138.55 138.92 (2C), 159.20; HRMS (ESI-TOF) *m/z* calcd for $C_{65}H_{62}F_{17}N_3O_9SNa$ $[M+Na]^+$ 1406.3833, found1406.3831.

General procedure for the synthesis of compounds 42-49. These compounds were prepared from corresponding azides (**33-41**) in a two-step sequence as describe below. The azides were converted to amines by Staudinger reduction, and the resultant amines were subsequently reacted with 9-different fatty acids, respectively. In a typical procedure, an azide (1.0 eq.) was dissolved in THF/H_2O (1 mL, 1:1 v/v) to which a 1M solution of PMe_3 in THF was added (2.5

eq.). After being stirred at rt for 12 h or until the azide was completely consumed, the solution was evaporated under reduced pressure. The residue was subjected to a FSPE cartridge to get the desired amine. A solution of amine (1 eq.) in anhydrous DMF (3 mL) was treated with acid (1.5 eq.), HBTU (1.2 eq.), and *N,N*-diisopropylethylamine (1.5 eq.) at 0 °C. The reaction mixture was stirred at rt for 16 h or until TLC indicated the disappearance of amine, then concentrated under reduced pressure and extracted with EtOAc (3 x 10 mL). The combined organic extracts were washed with brine, dried over $MgSO_4$, and concentrated in *vacuo*. The residue was purified by a FSPE cartridge (70% aqueous MeOH to 100% MeOH elution) to afford pure amides (**42-49)** (82-97% yield for 2-steps). The homogeneity of the compounds was verified by HR-ESI MS analysis. Isolated yields and observed m/z values are shown in parentheses (Table S2).

References

1. Plettenburg, O.; Bodmer-Narkevitch, V.; Wong, C. H. *J. Org. Chem.* **2002,** *67*, 4559.
2. Chang, C. W.; Chen, Y. N.; Adak, A. K.; Lin, K. H.; Tzou, D. L. M.; Lin, C. C. *Tetrahedron* **2007,** *63* (20), 4310-4318.
3. Huang, L. D.; Lin, H. J.; Huang, P. H.; Hsiao, W. C.; Reddy, L. V.; Fu, S. L.; Lin, C. C. *Org. Biomol. Chem.* **2011**, *9*, 2492-2504.
4. Moumé-Pymbock, M.; Furukawa, T.; Mondal, S.; Crich, D. *J. Am. Chem. Soc.* **2013**, *135*, 14249−14255.
5. Morita, M.; Motoki, K.; Akimoto, K.; Natori, T.; Sakai, T.; Sawa, E.; Yamaji, K.; Koezuka, Y.; Kobayashi, E.; Fukushima, H. *J. Med. Chem.* **1995**, *38*, 2176.
6. Fujio, M.; Wu, D.; Garcia-Navarro, R.; Ho, D. D.; Tsuji, M.; Wong, C. H. *J. Am. Chem. Soc.* **2006,** *128*, 9022.
7. Chang, Y. J.; Huang, J. R.; Tsai, Y. C.; Hung, J. T.; Wu, D.; Fujio, M.; Wong, C. H.; Yu, A. L. *Proc. Nat. Acad. Sci.* **2007,** *104*, 10299
